# Genomic prediction accuracy for switchgrass traits related to bioenergy within differentiated populations

**DOI:** 10.1186/s12870-018-1360-z

**Published:** 2018-07-09

**Authors:** Jason D. Fiedler, Christina Lanzatella, Serge J. Edmé, Nathan A. Palmer, Gautam Sarath, Rob Mitchell, Christian M. Tobias

**Affiliations:** 1 Department of Plant Sciences, North Dakota State University, 166 Loftsgard Hall, Fargo, ND 58108-6050 USA; 2 USDA-ARS Crop Improvement and Genetics Research Unit, Western Regional Research Center, Albany, CA 94710 USA; 3 USDA-ARS Wheat, Sorghum and Forage Research Unit, 251 Filley Hall/Food Ind. University of Nebraska, East Campus, Lincoln, NE 68583 USA

**Keywords:** Perennial, Biomass, Biofuel, Panicum, Polycross

## Abstract

**Background:**

Switchgrass breeders need to improve the rates of genetic gain in many bioenergy-related traits in order to create improved cultivars that are higher yielding and have optimal biomass composition. One way to achieve this is through genomic selection. However, the heritability of traits needs to be determined as well as the accuracy of prediction in order to determine if efficient selection is possible.

**Results:**

Using five distinct switchgrass populations comprised of three lowland, one upland and one hybrid accession, the accuracy of genomic predictions under different cross-validation strategies and prediction methods was investigated. Individual genotypes were collected using GBS while kin-BLUP, partial least squares, sparse partial least squares, and BayesB methods were employed to predict yield, morphological, and NIRS-based compositional data collected in 2012–2013 from a replicated Nebraska field trial. Population structure was assessed by *F* statistics which ranged from 0.3952 between lowland and upland accessions to 0.0131 among the lowland accessions. Prediction accuracy ranged from 0.57–0.52 for cell wall soluble glucose and fructose respectively, to insignificant for traits with low repeatability. Ratios of heritability across to within-population ranged from 15 to 0.6.

**Conclusions:**

Accuracy was significantly affected by both cross-validation strategy and trait. Accounting for population structure with a cross-validation strategy constrained by accession resulted in accuracies that were 69% lower than apparent accuracies using unconstrained cross-validation. Less accurate genomic selection is anticipated when most of the phenotypic variation exists between populations such as with spring regreening and yield phenotypes.

**Electronic supplementary material:**

The online version of this article (10.1186/s12870-018-1360-z) contains supplementary material, which is available to authorized users.

## Background

The ability to accurately predict performance and breeding values of crops and livestock based on large numbers of genetic markers in the absence of phenotypic data has been the focus of intense research efforts and is now being applied in advanced breeding programs [18, 35, 56]. This approach, known as genomic selection (GS), would be useful, in theory, for perennial grasses such as switchgrass that require 2–3 seasons to determine their full yield potential. In dairy cattle, the generation interval has been reduced from 4 to 7 y to approximately 2.5 y, allowing annual rates of genetic gain to increase from ~ 50–400% depending on the heritability of the trait [18]. In crops such as maize and wheat, predictions show that GS accuracies as low as 0.2 can achieve greater annual genetic gains than marker-assisted selection breeding schemes [24]. In perennial species, annual genetic gains and cost per unit of gain are also predicted to be higher with GS [20, 48, 64]. This can be the case with switchgrass (*Panicum virgatum L.*) if genomic estimates of breeding values (GEBV) are sufficiently accurate and the generation interval can be shortened.

Switchgrass is an attractive, perennial biomass crop for the US, due to its favorable net energy yield as well as several environmental and economic advantages [51]. Switchgrass is open pollinated and highly self-incompatible with ample genetic diversity [53]. The genetic variation between cultivars can differ 3–5 fold for biomass [54], 10% for lignin content [4], and 85% for water-use efficiency [28]. Some switchgrass lines reach heights of more than 3 m, and have vigorous root systems that may extend to depths of more than 3.5 m [41]. Phenotypic variation in many traits is sufficient for genetic analysis and breeding. Breeding programs have focused on recurrent selection of synthetic cultivars from wild switchgrass populations, targeting traits such as (1) high biomass yields, (2) improved seedling establishment, and (3) increased feedstock quality [3]. In the upper southeastern US, switchgrass biomass yields average more than 14 Mg ha^− 1^ [17], and in some locations more than 25 Mg ha^− 1^, suggesting greater yield potential with further improvement by breeding.

Defining the type of selection scheme and relative advantage of implementing GS in forages, it was found that under certain scenarios GS could be advantageous over phenotypic selection. For allogamous species such as switchgrass the GS advantage can result in increases of up to 2.6 fold [52].

To date, genomic prediction in switchgrass has been attempted in panels of northern-adapted upland and lowland germplasm and in two populations of half-sib families, one upland and one derived from the Liberty cultivar [5, 31, 47]. These reports employed a variety of different modeling and genotyping methods and represented different population structures and unique environments. Reported accuracies have been from over 0.5 to negative ranges depending on the trait, environment, and composition of the calibration set (CS) and validation set (VS).

The composition of the CS has been shown to be critical for improving the accuracy of GS, particularly under situations where there is population structure [26]. Population structure can produce false associations in genome-wide association studies [45, 66]. It can introduce upward bias to estimates of heritability [58], and can introduce bias into genomic prediction [32, 49, 65]. Capturing as much phenotypic variation as possible in the CS is desirable [26]. However, significant population structure should be accounted for when evaluating performance of the CS and genomic prediction accuracies decline as the relationship between CS and VS becomes weaker [23].

In this study, the impact of population structure on estimations of genomic heritability and on genomic prediction was assessed using both compositional and morphological traits in switchgrass related to bioenergy. The genetic trials were conducted with space planted populations with unknown pedigree relationships. These populations were derived from both upland and lowland ecotypes and therefore represent high diversity. Four different methods for genomic prediction were tested and their performance compared. Heritability was partitioned into across and within-population components that may have specific contributions to prediction accuracy.

## Methods

### Plant populations

Experiments were established from greenhouse-grown seedlings in 2009 in the field at the Eastern Nebraska Research and Extension Center (ENREC) near Mead, NE. One hundred twenty-five seedlings were planted for each of five populations in 5 rows of 25 seedlings on 1.1 m centers. Populations were randomly assigned to plots and genotypes within populations were randomly assigned to individual rows and positions within the plots. No fertilizers were applied in the establishment year while in post-establishment years 112 kg ha^− 1^ nitrogen was applied in the spring as NH_4_NO_3_. In establishment and post-establishment years herbicides and hand-weeding were used to control annual weeds. Atrazine (6-chloro-*N*-ethyl-*N*-isopropyl-1,3,5,-triazine-2,4-diamine), 2,4 -D (2,4-dichlorophenoxyacetic acid) and S-metolachlor [2-chloro-*N*-(2-ethyl-6-methylphenyl)-*N*-(2-methoxy-1-methyl) acetamide] were applied at rates of 1.96, 1.48, and 1.26 kg ha^− 1^ respectively once in late spring or early summer. In 2011 two ramets were dug from each genotype in the original plots and used to establish two additional randomized plots per population, keeping the populations separate blocks. Residual plot stubble was burned in April 2011 and again in April 2012 prior to the onset of spring regrowth.

The five populations used in this study were either obtained from NRCS Plant Material Centers or were harvested from isolated polycross blocks near Mead, NE which were maintained at least 500 m from other switchgrass fields. All populations were assumed to be tetraploid based on breeding history. A summary of the populations is presented in Table 1. Kanlow (K) is a released tetraploid lowland cultivar with high yield. Kanlow N1 (N1) has been previously described [59] and represents a population that had survived 4 years without winter kill in Nebraska. Summer (S) is a tetraploid, upland cultivar adapted to more northern latitudes with good winter survival. The seed lot used to establish our Summer population represented a bulk harvest of a selection nursery comprised of 1000 individuals with high yield and good vigor. The Kanlow N1-EM (N1EM) population is an early flowering population of plants selected from the Kanlow N1 base. The Kanlow x Summer F2 (KxS) population represents a population two generations removed from the initial randomly intercrossed parental populations in which Kanlow was used as the male parent.

**Table 1:** Switchgrass seed sources used in this study

Strain	Description	Source
‘Kanlow’ (K)	Released lowland-tetraploid cultivar	NRCS-Manhattan Kansas Manhattan Plant Materials Center
Kanlow N1 (N1)	Kanlow-derived population that had survived winter-kill over a four year period in Nebraska	Syn 2 increase of isolated polycross from selected plants
‘Summer’ (S)	Upland tetraploid with exceptional vigor and biomass yield in Nebraska and heading later than DOY 209 in 2005	Syn 1 increase from 1000 individual polycross nursery
Kanlow N1 Early Maturity (N1EM)	Derived from Kanlow N1 with high yields and heading day earlier than DOY224	Syn 1 bulk harvest from 15 individuals grown in an isolated polycross nursery
KxS F2 (KxS)	Upland-lowland hybrid population, K(♂) × S(♀)	Syn 2 seed increase 1 generation removed from initial hybrids grown in an isolated polycross

### Phenotypic analysis

#### Spring emergence

Spring emergence (GRN) was determined in 2013 as the day of year (DOY) when green shoots visibly emerged from winter dormancy.

#### Yield

All plots were harvested on a single clone basis after a killing frost between November 2 and 7, 2012 using a Carter Harvester (Carter Manufacturing Co., Brookston, IN) set at a cutting height of 10 cm. Fresh weights of individual clones were determined and subsamples were taken to determine percent moisture and provide an estimate of dry weight. Yield (YLD) was reported as dry weight corrected for the weight of the subsample.

#### Anthesis

The anthesis date (ANT) was determined as the day of year when 50% of the mature tillers of each clone reached the anther emergence/anthesis R4 stage [39].

#### Near infrared spectroscopy data

Harvested samples were dried in a forced air oven at 60 °C. These samples were then ground in a Wiley mill to pass a 2 mm test sieve and later in a Cyclone Mill to pass a 1 mm test sieve. Ground samples were scanned using a Model 6500 near-infrared spectrometer (FOSS NIRSystems, Inc., Laurel, MD). Estimates of over 20 biomass components, including theoretical yields of simple sugars and ethanol, were obtained from broad-based near infrared spectroscopy (NIRS) calibrations. Descriptions of the traits can be found in Table 2 and in more detail in [61].

**Table 2:** Repeatability () whole plant, biomass composition, and actual and potential ethanol yield traits among the populations tested

Variable	Abbreviation	Units	${\widehat{H}}^2$	Population				
				K	KxS	N1	N1EM	S
Whole plant								
Dry matter Yield	YLD	kg	0.61	1.45 ± 0.43a^a^,^b^	1.03 ± 0.31c	1.23 ± 0.32b	1.37 ± 0.36a	0.6 ± 0.22d
Anthesis Date	ANT	DOY	0.94	246.0 ± 4.0a	227.4 ± 10.4d	240.8 ± 8.6b	235.2 ± 7.5c	202.0 ± 10.5e
Spring Emergence Date	GRN	DOY	0.78	137.6 ± 8.7a	133.1 ± 1.5c	138.3 ± 1.5a	137.7 ± 1.2b	130.7 ± 1.9d
Composition								
Dry Matter	DM	mg g^− 1^	0.47	911.4 ± 1.9a	911.2 ± 2.1a	910.4 ± 1.6bc	911.0 ± 1.9ab	910.2 ± 2.5c
Carbon	C	mg g^− 1^	0.71	448.9 ± 3.1a	445.6 ± 3.2c	447.53 ± 2.9b	450.0 ± 3.2a	444.0 ± 3.9d
Nitrogen	N	mg g^− 1^	0.6	5.76 ± 1.03ab	5.27 ± 0.94c	5.92 ± 0.87a	5.47 ± 0.97bc	5.57 ± 1.16abc
Extracted fat	FAT	mg g^− 1^	0.71	9.23 ± 1.22b	7.12 ± 1.28c	9.59 ± 1.23ab	9.7 ± 1.1a	7.32 ± 1.23c
Minerals (total ash)	ASH	mg g^− 1^	0.72	63.12 ± 5.6b	71.31 ± 7.38a	62.47 ± 5b	61.1 ± 5.1b	70.11 ± 8.64a
Klason Lignin	KL	mg g^− 1^	0.46	286.4 ± 16.0c	298.5 ± 15.0a	287.1 ± 12.6c	293.3 ± 12.7b	296.6 ± 15.1ab
Uronic Acids	UA	mg g^− 1^	0.67	17.04 ± 0.36b	16.74 ± 0.39c	17.2 ± 0.35a	17.25 ± 0.4a	16.39 ± 0.48d
Rhamnose	RHA	mg g^− 1^	0.49	1.70 ± 0.14d	1.75 ± 0.16bc	1.77 ± 0.13b	1.7 ± 0.12 cd	1.85 ± 0.21a
Fucose	FUC	mg g^− 1^	0.54	0.23 ± 0.04b	0.26 ± 0.03a	0.22 ± 0.03c	0.23 ± 0.03b	0.23 ± 0.04b
Arabinose	ARA	mg g^− 1^	0.49	32.93 ± 1.48b	33.35 ± 1.44ab	33.76 ± 1.41a	32.94 ± 1.31b	33.79 ± 1.65a
Xylose	XYL	mg g^− 1^	0.66	212.4 ± 5.7c	214.8 ± 6.4b	212.3 ± 6.3c	212.9 ± 6.1bc	218.3 ± 7.1a
Mannose	MAN	mg g^− 1^	0.59	8.95 ± 0.83ab	7.82 ± 0.85c	8.79 ± 0.74a	9.10 ± 0.87b	8.71 ± 1.41d
Galactose	GAL	mg g^− 1^	0.41	9.61 ± 0.67b	9.63 ± 0.74b	9.96 ± 0.62a	9.53 ± 0.53b	9.7 ± 0.88ab
Glucose	GLC	mg g^− 1^	0.47	298.1 ± 7.2b	296.6 ± 7.2b	295.4 ± 6.9b	301.0 ± 6.1a	296.0 ± 8.0b
p-Coumarate esters	PCA	mg g^− 1^	0.83	8.32 ± 0.55a	7.53 ± 0.69c	8.06 ± 0.57b	8.01 ± 0.59b	6.46 ± 0.75d
Esterified ferulates	FEST	mg g^− 1^	0.9	1.91 ± 0.15a	1.53 ± 0.23c	1.86 ± 0.18ab	1.81 ± 0.18b	1.2 ± 0.2d
Etherified ferulates	FETH	mg g^−1^	0.34	1.05 ± 0.2a	0.82 ± 0.26bc	0.92 ± 0.19b	1.05 ± 0.19a	0.82 ± 0.26c
Cell wall concentration	CWC	mg g^− 1^	0.62	825.0 ± 20.0d	850.2 ± 19.6b	828.8 ± 19.4d	840.7 ± 17.0c	860.1 ± 21.3a
ARA + XYL + Man+GAL	AXMG	mg g^− 1^	0.7	259.8 ± 5.9b	261.2 ± 6.5b	260.4 ± 6.8b	261.0 ± 6.1b	266.5 ± 6.4a
ARA + XYL	AX	mg g^− 1^	0.73	240.1 ± 5.3c	245.5 ± 5.8b	241.3 ± 6.4c	241.5 ± 5.6c	252.1 ± 6.5a
Sucrose	SUC	mg g^−1^	0.81	14.77 ± 4.72a	8.94 ± 4.37c	16.30 ± 4.79a	12.83 ± 3.50b	5.65 ± 3.95d
Soluble glucose	GLCS	mg g^− 1^	0.81	10.50 ± 1.92ab	8.30 ± 1.93c	11.10 ± 1.88a	10.21 ± 1.67b	6.85 ± 2.01d
Fructose	FRU	mg g^− 1^	0.75	8.83 ± 2.25b	6.57 ± 2.05c	9.75 ± 2.46a	8.20 ± 1.96b	5.21 ± 2.10d
Total soluble carbohydrates	SC	mg g^− 1^	0.85	34.65 ± 8.60a	22.75 ± 7.85c	36.32 ± 8.61a	29.4 ± 6.76b	17.66 ± 7.39d
Starch	STA	mg g^−1^	0.55	8.11 ± 1.80ab	7.73 ± 2.60b	7.71 ± 2.06b	7.95 ± 1.73b	8.88 ± 2.20a
Non-structural carbohydrates (starch + SC)	NSC	mg g^− 1^	0.82	33.63 ± 10.14a	21.28 ± 9.75c	35.12 ± 10.35a	29.13 ± 7.59b	16.95 ± 9.04d
Total hexoses	HEX	mg g^− 1^	0.82	373.6 ± 9.7a	359.8 ± 10.2b	372.7 ± 7.7a	375.1 ± 7.2a	348.4 ± 10.8c
Total sugars	SUG	mg g^− 1^	0.62	664.8 ± 10.1b	658.2 ± 10.9c	666.7 ± 10.0ab	668.8 ± 10.0a	658.7 ± 11.6c
Ethanol and potential ethanol								
Ethanol/g dry forage	ETOH	mg g^− 1^	0.81	79.79 ± 5.24ab	73.41 ± 5.83c	81.65 ± 6.06a	79.22 ± 4.98b	70.07 ± 6.02d
Pentose sugars relased/g dry forage	PENT	mg g^−1^	0.68	213.0 ± 5.2a	217.3 ± 6.8b	213.9 ± 5.9a	214.3 ± 6.0a	223.8 ± 7.4c
Proportion of hexoses that are non-structural or soluble	PSOL	%	0.8	0.09 ± 0.03a	0.06 ± 0.02c	0.09 ± 0.03a	0.07 ± 0.02b	0.05 ± 0.02d
Pentose proportion of total carbohydrates	PPEN	%	0.84	0.43 ± 0.01d	0.44 ± 0.01b	0.43 ± 0.01c	0.43 ± 0.01 cd	0.45 ± 0.01a
Theoretical ethanol from hexoses (excluding starch)	HEXE	mg g^− 1^	0.71	202.7 ± 4.5b	198.4 ± 5.2c	203.6 ± 3.9ab	204.3 ± 3.9a	194.9 ± 6.4d
Estimated ethanol from non-structural carbohydrates	NSCE	mg g^− 1^	0.79	17.10 ± 4.64a	11.78 ± 4.74c	17.98 ± 4.76a	14.83 ± 3.64b	9.74 ± 4.36d
Cell wall ethanol	CWE	mg g^− 1^	0.68	63.06 ± 4.14b	59.61 ± 3.62c	64.64 ± 3.74a	63.76 ± 4.26ab	59.03 ± 3.84c
Theoretical ethanol conversion efficiency from cell wall hexosans	CWEP	%	0.48	35.28 ± 3.19b	34.42 ± 2.88b	36.23 ± 2.64a	35.10 ± 2.87b	34.35 ± 3.36b
Pentoses extraction efficiency	PENTP	%	0.6	78.28 ± 1.10b	78.53 ± 1.28b	78.93 ± 1.11a	78.40 ± 0.88b	79.41 ± 1.23a
Hexose ethanol extraction efficiency	HEXEP	%	0.68	42.02 ± 2.78b	39.54 ± 2.83c	43.38 ± 3.06a	41.56 ± 2.83b	37.30 ± 3.30d
Forage quality composition								
In vitro dry matter digestibility	IVDMD	mg g^− 1^	0.71	360.3 ± 21.9a	321.3 ± 22.1c	363.4 ± 22.3a	346.7 ± 19.7b	308.6 ± 27.5d
Neutral detergent fiber	NDF	mg g^−1^	0.44	778.5 ± 15.9c	790.2 ± 14.7ab	773.0 ± 15.9c	787.3 ± 14.4b	793.7 ± 18.9a
Acid detergent fiber	ADF	mg g^− 1^	0.49	418.3 ± 17.9c	431.8 ± 15.8a	409.6 ± 17.5d	421.6 ± 15.3bc	425.6 ± 21.3ab
Acid detergent lignin	ADL	mg g^− 1^	0.47	66.1 ± 4.2b	69.9 ± 4.0a	64.6 ± 3.9c	66.9 ± 4.0b	68.6 ± 5.8a
Total energy content	CAL	cal	0.63	4115 ± 10a	4095 ± 10d	4112 ± 12ab	4110 ± 11b	4106 ± 12c

^a^Average ± standard deviation^b^For each trait, different letters designate values that are significantly different from one another, α < 0.05

### Phenotypic data analysis

Estimates of best linear unbiased predictors (BLUP) values for each trait across replicates, genotypic variance (*σ*_G_^2^), error variance (*σ*_*e*_^2^), and repeatability (*H*^*2*^) were obtained using the restricted maximum likelihood method in the “lme4” package [2] in R (R Core Team). Broad-sense heritability, equivalent to line-based repeatability across plots, was estimated from the variance components obtained with the BLUP model:

$$ {H}^2=\frac{\sigma_G^2}{\sigma_G^2+\frac{\sigma_e^2}{n}}, $$

where *n* refers to the average number of replicates per genotype. Data was centered and scaled by the standard deviation prior to analysis to allow simpler comparisons among methods and traits and some outliers were removed.

### Genotyping

Leaf tissue samples from the original plots prior to establishing replicates were collected and genomic DNA was extracted using a CTAB method [6]. Genotyping-by-sequencing library production followed a previously established protocol [13] with a few modifications. Briefly, gDNA was digested with *Pst*I and ligated to Illumina adapters with a custom set of sequence index barcode tags. Barcoded DNA was subsequently adapted with Illumina sequencing primers and purified with a Qiagen PCR cleanup kit. DNA libraries were pooled (96 samples per flow-cell land) and single-end reads were generated on an Illumina HiSeq 2000 at the Vincent J. Coates Genomics Sequencing Laboratory, University of California, Berkeley.

Sequencing generated 1.25 × 10^9^ 100-bp single-end reads that were fed to the TASSEL-GBSv2 pipeline [19] and generated a total of 810 million 64-bp sequence tags of which 721 million could be matched to the master tag database aligned to version 4.0 of the switchgrass genome assembly (https://www.phytozome.jgi.doe.gov) using BWA [30]. For the alignment, only the 18 main chromosomes were used. Prediction and annotation of single nucleotide polymorphism (SNP) effects were performed using SNPEff [9]. The number of tags across individual taxons ranged from 13,777 to 5,104,802 with an average of 1,229,285. TASSEL detected a total of 70,215 SNP sites with 46% of potential genotype calls present.

SNP filtering was then performed. First, genotypes with a depth of 1 were set to missing which eliminated 2.33% of the individual SNP calls. Second, individuals were filtered out if they contained missing information for > 75% of the SNP positions. This resulted in the loss of 104 individuals (16% of the population). Third, sites with a minor allele frequency of < 0.025 and with genotype calls in fewer than 50% of all individuals were eliminated. Fourth, sites in complete LD with other sites were pruned. After applying these filters, a total of 19,342 SNPs remained across 483 individuals with 71% of the potential genotype calls present.

### Genetic differentiation and genomic heritability estimates

For evaluating genomic selection methods and heritability imputation of missing genotypes was necessary and was conducted using the LD-kNNi algorithm where the *l* sites most in LD with the site to be imputed are used to determine the nearest neighbors and the weightings to be used when imputing [38]. In the imputed dataset the parameters *k* = 13, *l* = 8 were applied. Accuracy was determined by masking and imputing 10,000 known genotypes and was estimated at 88.5%.

Population differentiation was measured on the imputed dataset with the *F*_*ST*_ statistic using the method of Weir and Cockerham [62]. Individual clustering by principal components analysis was performed with the genotypic data for the purposes of visualizing population structure [33].

For the purposes of predicting genomic breeding values (GEBV) and estimating genomic heritability within and across populations, additive genetic variance (*σ*_G_^2^) and residual variance (*σ*_*e*_^2^) were derived from applying ridge-regression mixed modeling kinship-BLUP used a restricted maximum likelihood (REML) approach implemented in the R package rrBLUP version 4.5 [14, 46]. The estimate of the realized additive relationship matrix was calculated as:

$$ \widehat{A}=\frac{W{W}^{\prime }}{2{\sum}_i{p}_i\left(1-{p}_i\right)} $$

where W is the centered (*n x m)* genotype matrix and *p* is the allele frequency [15]. The basic model

$$ y= X\beta + Zu+e $$

$$ u\sim N\left(0,K{\sigma}_g^2\right) $$

was used where **X** is a full-rank design matrix for the fixed effects (β), **Z** is the design matrix for the random effects **u**, **K** is the additive relationship matrix **A,** and *e* is for the residuals (e ~ *N* (0, ***I****σ*_*e*_^2^)) with **I** being the identity matrix.

The matrix **K** captures population structure due to admixture, genetic drift, and selection history as well as kinship. Here, marker-based estimates of heritability across and within subpopulations (*h*_*g*_^2^, *h*_*gA*_^2^*, h*_*gW*_^2^) were derived based on the BLUP phenotypes and on orthogonal decomposition of the genetic variance components by reparameterizing the basic model above. This was accomplished in a manner similar to that of Guo et al. [21] based on initial work of [12, 27]. After eigenvalue decomposition of K as

$$ K={UDU}^{\prime } $$

where **U** is an *n* x (*n*-1) matrix of the eigenvectors of **K** with **U**_i_ the column I (*i* = 1,2, …, n-1) representing the individual principal component loadings, and **D** is an (*n*-1) x (*n*-1) diagonal matrix with each diagonal element representing eigenvalues of **K**, arranged in decreasing value λ_1_, λ_2_, . λ_n - 1_. The basic model is then rewritten as

$$ y=1\mu + U\alpha +e $$

and **α** becomes an (*n*-1) × 1 vector of random genetic effects that have a normal distribution *N* (**0**, **D**σ_**G**_^**2**^) with the principal components used as random variables that has the same distribution as the basic model yet allows separation of subpopulation structure explained by the dominant principal components (*d*) into across-population genetic variance,

$$ {\sigma}_{gA}^2=\frac{1}{n-1}\sum \limits_{i=1}^d{\alpha}_i^2 $$

and within-population genetic variance,

$$ {\sigma}_{gW}^2=\frac{1}{n-1}\sum \limits_{i=d+1}^{n-1}{\alpha}_i^2 $$

The total genetic variance was calculated as

$$ {\sigma}^2=\frac{1}{n-1}\sum \limits_{i=1}^{n-1}{\alpha}_i^2 $$

Corresponding marker-based trait heritability were obtained as [27]:

$$ {h}_{gA}^2=\frac{\sigma_{gA}^2}{\sigma^2+{\sigma}_e^2} $$

$$ {h}_{gW}^2=\frac{\sigma_{gW}^2}{\sigma^2+{\sigma}_e^2} $$

$$ {h}_g^2=\frac{\sigma^2}{\sigma^2+{\sigma}_e^2} $$

### Genomic prediction

Four methods were used to model the data: (1) a kinship-BLUP method (kin-BLUP); (2) a partial least squares regression method (PLS), (3) a sparse, partial least squares method (SPLS), and (4) BayesB (BB).

Prediction accuracy *r* (correlation of the VS GEBV with their BLUP values) was assessed for all methods using modified 5-fold cross-validation (CV) where the VS was constructed from specific populations or combinations of populations and divided into 5 sets that each comprised a single fold. The calibration set (CS) then consisted of the remaining individuals from all populations. Initial broad screening of all traits using 5-fold CV across the entire dataset (“All”) enabled us to focus on traits with higher prediction accuracy. In general, analysis was limited to those 22 traits with prediction accuracies greater than 0.5 with the kin-BLUP method.

Each analysis, except for BayesB, was conducted using 20 replications of a 5-fold CV approach to estimate prediction accuracy within a single population or combination of populations. The subjects were divided at random into five sets. For each set, the phenotypic BLUP values (*y*) were sequentially used as a validation set (VS) by masking and predicting the values $\widehat{y}$ based on the model parameters estimated with the other four sets and with inclusion of other genotypes from outside the subject group that served as the VS. The CS always included all individuals not included in the VS.

Accuracy was determined from the average phenotypic correlation $r\left({\widehat{y}}^2,{y}^2\right)$ between the true phenotype (*y*) and GEBV $\left(\widehat{y}\right)$ across folds and replications. The mean squared error (MSE) was estimated from $\frac{1}{n}\sum {\left(y-\widehat{y}\right)}^2$ where *n* is the number of individual VS genotypes used for each fold. Reported MSE values were averaged across folds and replicates.

#### Partial least squares method

The partial least squares method (PLS) introduced by Wold [63] decomposes the genotypic data **W** in orthogonal scores T called latent variables and loadings P that are obtained by projecting the predicted variables and the observable variables to a new space and regressing *y* not on **W** but on the first *a* columns of the scores **T**. The latent variables are obtained individually in an iterative process which are then used as new variables of a linear regression. This allows to account for collinearity among the predictor variables and instances with many more predictor variables than observations. The PLS method was implemented with the R packages ‘pls’ [36]. In this specific instance the first 15 latent variables were used for prediction.

#### Sparse partial least squares method

The sparse partial least squares method (SPLS) [29], like PLS, allows selection of a sparse set of explanatory variables and dimensionality reduction while maintaining good predictive ability. In the SPLS case, this is done through imposing constraints onto the explanatory variables that are included in the loadings P. The constraints are imposed through a threshold parameter λ_1._ Values for this parameter as well as the optimal number of latent variables to use for each trait were obtained through a cross-validation approach. SPLS prediction was implemented using the ‘SPLS’ package [7, 8].

#### BayesB method

This method allows a portion (π) of the markers to have large effects while most markers have no effects. Marker variance is unique for each marker. Marker effects were estimated with Monte Carlo Markov Chan simulations. The BayesB model was run with the ‘BGLR’ package in R that employs a Gibbs sampling algorithm [11, 35]. A value of 0.05 for π was used with 5000 iterations and a 1500 iteration burn-in period and a sampling frequency of 5.

## Results

### Read distribution and SNP diversity

The sequence tags aligned to a total of 148,557 unique genome loci or approximately 104 Mbp^− 1^, of which 19,769 (13%) overlapped or intersected with annotated gene regions. This is comparable to the 15% of the total switchgrass genome annotated as gene region and demonstrates little ascertainment bias using GBS. Within these sequences, 19,342 SNPs were identified after filtering. These were primarily biallelic and are summarized in Table 3. Of the 19,342, 52.1% were transitions and 44% were transversions (TsTv = 1.183), 0.8% were single nucleotide indels, 3.6% were triallelic, and 0.1% were tetra-allelic. On average, there were 14.84 SNP Mbp^− 1^ detected with a strong significant relationship between the total number of SNPs and the annotated length of the chromosome (*P* < 0.001, Adjusted *R*^*2*^ = 0.7791), and between SNP density and distance to the end of the chromosomes (*P* < 0.001, Adjusted *R*^*2*^ = 0.7147). A total of 5118 SNPs were annotated in coding sequences with 65% of the non-reference alleles caused by either missense or nonsense mutations, 2216 were in introns in gene regions, 786 were annotated in the 3’ UTR region, and 670 were annotated in the 5’ UTR region.

**Table 3:** Characterization of SNP obtained in five populations by GBS after alignment with the switchgrass genome v. 4.1 and filtering

Parameter	value
Ts:Tv ratio	1.183
1 bp indel	0.8%
tri- + tetra-allelic	3.7%
Average SNP Mbp^− 1^	14.84
Annotated coding	5118
^a^Missense	3275 (64%)
Silent	1740 (34%)
Nonsense	76 (1.4%)
introns	2216
3’UTR	786
5’ UTR	670
Total SNPs	19,342

^a^Missense, silent, and nonsense were relative to reference AP13 genotype

### Differentiation of the populations

Population means and repeatability values for whole plant measurements and NIR traits are shown in Table 2. MANOVA tests demonstrated significant influences of population of origin on both morphological and NIR-compositional data. Population yield averages ranged from 0.6 kg dry matter YLD for Summer to 1.47 kg for Kanlow. Timing of anthesis and spring emergence were different for each population. Anthesis ranged from July 20 (202 DOY) for Summer to September 2 (246 DOY) for Kanlow while spring regreening ranged from May 10 (130 DOY) for Summer to May 17 (137 DOY) for Kanlow. Individually, the Pearson product-moment correlation coefficient *r* between YLD and GRN was 0.22 while *r* between YLD and ANT was 0.43 (Additional file 1: Figure S1). There were also significant positive correlations within two groups of NIR-estimated cell wall properties (Additional file 1: Figure S2). These two groups were: (1) GLC, KL, ADL, NDF, DM, FUC; and (2) N, IVDMD, MAN, UA, RHA, ARA, GAL. There were significant negative correlations between these two groups of properties. Repeatability which, in this case, represents an upper limit of broad-sense heritability ranged from 0.94 for ANT DOY to 0.34 for etherified ferulates (FETH).

Population fixation statistics (*F*_*ST*_) were evaluated over all SNPs weighted by the population size and are reported in Table 4. Most of the genetic variation that can be explained by population structure is due to differences between the K, N1, and N1EM populations (as a group) and the S populations (*F*_*ST*_ ~ 0.39–0.40). The K, N1, and N1EM populations were all closely related (*F*_*ST*_ ~ 0.01–0.03) to each other. The hybrid-based KxS population share more similarities with both S and K-based populations (*F*_*ST*_ ~ 0.13–0.16) than these were to one another,. This indicated that there was significant genetic differentiation between the lowland-derived (K, N1, N1EM) and the upland (S) populations.

**Table 4:** *F*_*ST*_ estimates for pairs of switchgrass populations weighted by sample size (19,342 SNPs)

Comparison	*F*_*ST*_	Standard error
K v. S	0.3952	1.3 × 10^−3^
K v. N1	0.0131	1.1 × 10^−4^
K v. N1EM	0.0266	1.6 × 10^−4^
K v. KxS	0.147	6.4 × 10^−4^
S v. N1	0.3861	1.3 × 10^−3^
S v. N1EM	0.3949	1.3 × 10^−3^
S v. KxS	0.1559	7.6 × 10^−4^
N1 v.N1EM	0.0151	1.1 × 10^−4^
N1 v.KxS	0.1345	6.3 × 10^−4^
N1EM v. KxS	0.1446	6.6 × 10^−4^

Comparing the K, N1, and N1EM populations as a group with the S population, no fixed differences (*d*_*f*_) were found whereby the lowland populations would be homozygous for one allele and the upland population homozygous for another. However, 3715 sites were fixed in either group and segregating in the other.

Before performing the genomic predictions, the missing data was imputed using a k-nearest neighbor imputation method (LD-kNNi) for unordered markers. This approach uses pair-wise LD information from the markers that are the most tightly linked (i.e. in LD) in the genotypes to be imputed. Imputation resulted in 3,139,549 genotypes being added or 28% of the dataset. Prediction accuracy of the missing data was estimated to be 88.5% based on masking and imputing 10,000 known genotypes.

**Fig. 1:**
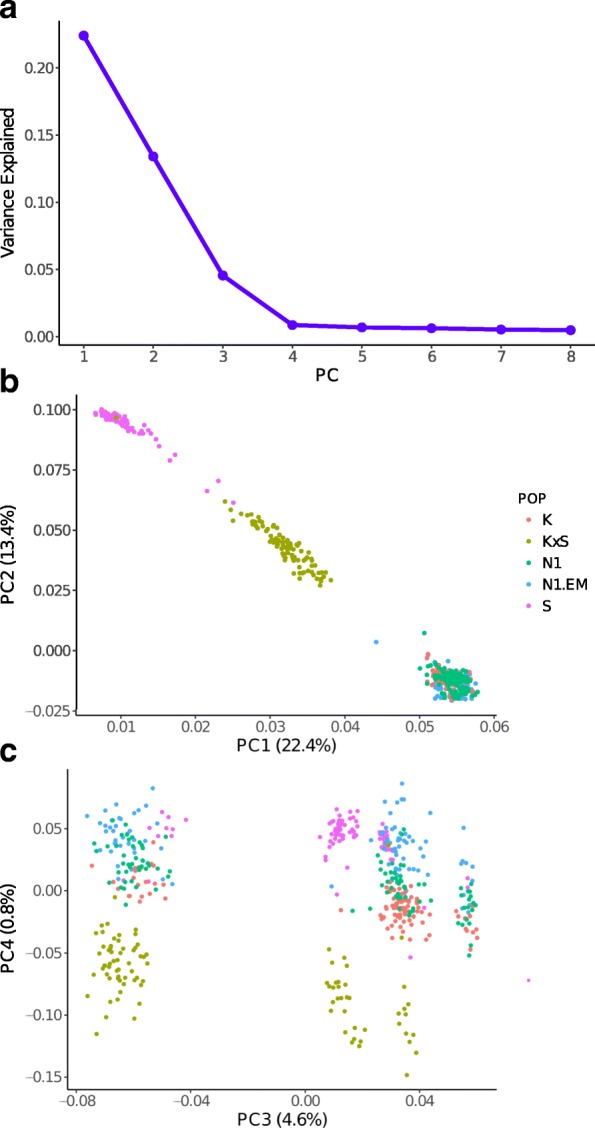
**a** Proportion of genetic variance explained by principal components (PC) 1 through 8. **b** Plot of PC loadings along axes (PC1) and (PC2) for individuals in the five switchgrass populations indicated. **c** Plot of PC loadings along axes (PC3) and (PC4) for the same individuals. Individuals are colored according to population as indicated in the legend for (**b**), and the percentage variance explained by each PC are indicated within parenthesis on each axis

Inference of population structure in the five populations based on PCA of the molecular data showed that the first three principal components accounted for 4.6–22.4% of the variation to jointly explain 40.4% (Fig. 1a). The populations can be completely resolved by the first PC (Fig. 1b, c) into three clusters represented by the lowland accessions (K, N1, and N1EM), the upland accession (S), and the hybrid accession (KxS) that is positioned between the two other groups. This result agrees with the group means of many of the phenotypes that indicate significant differences between these three populations (Table 2 and Additional file 1: Figure S3) as well as with the populations fixation statistics. Previous studies have also reported genetic differentiation between upland and lowland populations [16, 37, 40].

The first three PC were used to assess the impact of population structure on genomic heritability (*h*_g_^2^) for the entire data set by orthogonal decomposition of the genetic variance components. Separate genomic heritability estimates (*h*_g_^2^, *h*_gA_^2^, and *h*_gW_^2^) for selected traits are presented in Table 5 and the variance estimates are provided in Additional file 1: Table S5. Estimates for *h*_g_^2^ ranged from 0.57 for ANT and SC to 0.15 for YLD. The highest estimate for *h*_gA_^2^ was 0.43 for ANT while the lowest was 0.14 for ASH and YLD. More important for the accuracy of genomic selection applications, *h*_gW_^2^ estimates ranged from 0.01 for YLD to 0.31 for NSC. The *h*_gW_^2^ for ASH represented 62% of the total while the *h*_gW_^2^ for NSCE represented 60% of the total. Collectively, the phenological traits (ANT and GRN) and YLD were most influenced by population structure while the NIR-compositional traits were influenced to a variable degree. The *h*_gA_^2^ for NIR compositional traits ranged from 38 to 74% of total heritability.

**Table 5:** Trait dependent across and within-population genomic heritability estimates from switchgrass traits based on the first three principal components

Trait	^a^ *h*_*gA*_^*2*^	*h*_*gW*_^*2*^	*h*_*g*_^*2*^
ANT	0.43 ^b^(0.76)	0.14 (0.24)	0.57
ASH	0.14 (0.38)	0.23 (0.62)	0.38
AX	0.29 (0.74)	0.1 (0.26)	0.39
ETOH	0.26 (0.51)	0.25 (0.49)	0.51
FAT	0.16 (0.5)	0.16 (0.5)	0.32
FEST	0.34 (0.65)	0.18 (0.35)	0.51
FRU	0.26 (0.64)	0.15 (0.36)	0.41
GLCS	0.31 (0.63)	0.18(0.37)	0.49
GRN	0.46 (0.87)	0.03 (0.13)	0.49
HEX	0.26 (0.74)	0.09 (0.26)	0.35
HEXE	0.18 (0.57)	0.13 (0.43)	0.31
HEXEP	0.16 (0.48)	0.17 (0.52)	0.33
IVDMD	0.19 (0.65)	0.10 (0.35)	0.29
NSC	0.25 (0.45)	0.31 (0.55)	0.55
NSCE	0.20 (0.4)	0.30 (0.6)	0.50
PCA	0.25 (0.59)	0.17 (0.41)	0.42
PPEN	0.31 (0.67)	0.16 (0.33)	0.47
PSOL	0.25 (0.47)	0.29 (0.53)	0.54
SC	0.29 (0.51)	0.27 (0.49)	0.57
SUC	0.28 (0.59)	0.19 (0.41)	0.47
UA	0.18 (0.7)	0.08 (0.3)	0.26
YLD	0.14 (0.92)	0.01 (0.08)	0.15

^a^*h*_*gA*_^*2*^, across-population genomic heritability; *h*_*gW*_^*2*^, within-population genomic heritability; *h*_*g*_^*2*^, genomic heritability

^b^fraction of total genomic heritability in parentheses

### Genome-wide patterns of linkage disequilibrium

**Fig. 2:**
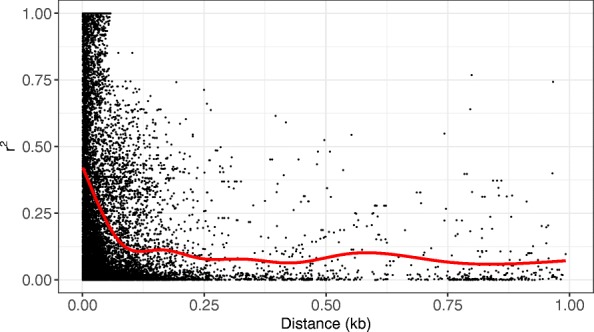
Genome-wide linkage disequilibrium. SNPs were evaluated for LD measured as *r*^*2*^ for patterns of linkage disequilibrium from pairs of SNPs separated by a 0.001–1 kb window. Smoothing (red line) was based on a penalized cubic regression spline

The accuracy of genomic selection is dependent on the degree of marker disequilibrium with underlying QTL for traits of interest. Marker data was used to model disequilibrium with potential QTL loci and calculated pairwise *r*^*2*^ for all markers positioned on the same chromosome. These were then sorted by distance and the results for the 0–1 kb distance interval are plotted in Fig. 2. The mean *r*^*2*^ was 0.178 within chromosomes across the entire dataset and appears to decay quite rapidly dropping to nominal levels within 0.25 kb.

Filtering the data, based on criteria including depth of sequence coverage required per genotype, data-missingness, or minor-allele frequency, had nominal effects on *r*^*2*^ values and resulted in loss of information as SNP sites were filtered out. Non-linear curve fitting using an exponential distribution was used to estimate the distance threshold for an *r*^*2*^ = 0.2. Changing the sequence depth required to call a genotype from 2 to 8 resulted in an approximate 6% increase in the threshold distance. Changing the amount of missing data allowed from 25 to 50% resulted in a 19% increase in threshold distance, while filtering sites based on minimum minor allele frequency from 0.05 to 0.20 resulted in a 380% increase in threshold distance. However, these changes only resulted in a small overall increase in threshold to 76 bp. The threshold for the S population was 69 bp while that for the combined lowland accessions (Low) was 58 bp. The KxS population had a lower threshold than either the S or K populations.

Because *r*^*2*^ is known to vary across the genome we attempted to look at trends across single chromosomes, but the data was too sparse to detect these variations reliably. Overall the threshold value ranged from 33 bp (Chr7N) to 82 bp (Chr3N) across all 18 chromosomes. Threshold values were not significantly different between the N and K subgenomes.

### Genomic prediction

Prediction accuracy results are summarized in Table 6 and Additional file 1: Tables S1-S4. In all cases, due to population structure, GEBVs were more accurate when the VS was sampled across the entire dataset (unconstrained). The highest accuracy using this CV method was obtained for Flowering DOY (ANT) using kin-BLUP (*r* = 0.88) while the following traits (FEST, HEX, PCA, PPEN, GRN, IVDMD, FAT, SUC, YLD, SC, GLCS, ETOH, HEXE, UA, NSC, FRU, ASH, AX, HEXEP, PSOL) were above 0.5 under the same conditions. When using a CV method where the VS was constrained to consist exclusively within one of either K, N1, N1EM, S, or KxS populations, ANT accuracy ranged from 0.46 in the N1 population to 0.06 in the K population and across different traits *r* ranged from 0.57 for Fructose (FRU) (MSE = 0.18) in the KxS population to − 0.23 (MSE = 0.05) for GRN in the KxS population. On average, across all traits and populations, accuracy was reduced 69.6% from the unconstrained CV strategy using kin-BLUP and similarly for the other prediction methods. GEBVs were slightly more accurate when the “Low” population was used as a VS rather than the individual K, N1, or N1EM populations and accuracies were reduced only 66.9% from the unconstrained CV strategy.

**Table 6:** Genomic prediction accuracy (*r*) for indicated traits using kin-BLUP, partial least squares (PLS), sparse partial least squares (SPLS), and BayesB (BB) regression methods and different cross-validation strategies

TRAIT	^a^VS	^b^*r* kin-BLUP	*r* PLS	*r* SPLS	*r* BB
ANT	All	^c^0.88	0.87	0.88	0.88
	K	0.06	− 0.06	0.02	−0.12
	KxS	0.23	0.24	0.23	0.34
	^d^Low	0.58	0.55	0.57	0.53
	N1	0.46	0.45	0.44	0.44
	N1EM	0.3	0.22	0.29	0.34
	S	0.11	0.11	0.12	0.23
ASH	All	0.58	0.58	0.59	0.62
	K	0.16	0.12	0.11	0.13
	KxS	0.26	0.27	0.28	0.22
	Low	0.2	0.2	0.22	0.09
	N1	0.15	0.16	0.17	0.19
	N1EM	0.16	0.21	0.24	0.28
	S	0.17	0.18	0.18	0.07
AX	All	0.58	0.56	0.58	0.52
	K	0.1	0.1	0.13	0.12
	KxS	0.26	0.26	0.08	0.19
	Low	0.14	0.14	0.05	0.02
	N1	0.1	0.19	−0.02	0.32
	N1EM	0.14	0.08	0.13	0.17
	S	0.13	0.12	0.09	0.05
ETOH	All	0.64	0.64	0.63	0.61
	K	0.2	0.31	0.24	0.31
	KxS	0.41	0.39	0.34	0.39
	Low	0.23	0.28	0.24	0.17
	N1	0.22	0.3	0.25	0.31
	N1EM	0.24	0.2	0.26	0.21
	S	0.18	0.2	0.14	0.06
FAT	All	0.68	0.68	0.68	0.71
	K	0.18	0.18	0.23	0.17
	KxS	0.1	0.06	0.11	0.14
	Low	0.37	0.4	0.38	0.34
	N1	0.41	0.51	0.47	0.35
	N1EM	0.4	0.42	0.38	0.44
	S	0.02	0.04	0.02	0.06
FEST	All	0.82	0.83	0.83	0.80
	K	0.08	0.07	0.14	0.26
	KxS	0.44	0.4	0.39	0.36
	Low	0.31	0.32	0.35	0.33
	N1	0.22	0.27	0.26	0.41
	N1EM	0.44	0.43	0.48	0.28
	S	0.37	0.48	0.5	0.42
FRU	All	0.59	0.59	0.59	0.56
	K	0.05	0.09	0.05	−0.06
	KxS	0.57	0.48	0.43	0.51
	Low	0.17	0.21	0.2	0.13
	N1	0.12	0.21	0.19	0.03
	N1EM	0.06	0.05	0.1	0.12
	S	0.1	0.11	0.15	0.27
GLCS	All	0.64	0.64	0.63	0.71
	K	0.06	0.05	0.01	0.2
	KxS	0.45	0.44	0.39	0.52
	Low	0.1	0.16	0.14	0.17
	N1	0.08	0.12	0.12	0.25
	N1EM	0.04	0.12	0.15	0.09
	S	0.24	0.36	0.32	0.35
GRN	All	0.75	0.65	0.88	0.88
	K	−0.05	−0.13	−0.04	0.2
	KxS	0	−0.28	0.03	−0.31
	Low	0.05	−0.1	0.12	0.04
	N1	0.19	−0.07	0.28	−0.08
	N1EM	0.01	−0.05	−0.02	0.01
	S	0.25	0.2	0.27	0.2
HEX	All	0.78	0.78	0.77	0.78
	K	0.27	0.21	0.21	0.22
	KxS	0.39	0.45	0.34	0.13
	Low	0.16	0.18	0.16	0.24
	N1	−0.02	0.08	0.09	0.16
	N1EM	0.15	0.19	0.24	0.18
	S	0.34	0.39	0.35	0.39
HEXE	All	0.64	0.65	0.65	0.60
	K	0.27	0.25	0.14	0.22
	KxS	0.39	0.46	0.41	0.5
	Low	0.18	0.21	0.2	0.24
	N1	−0.02	0.04	0.11	0.06
	N1EM	0.2	0.26	0.21	0.27
	S	0.26	0.31	0.29	0.34
HEXEP	All	0.58	0.59	0.58	0.53
	K	0.12	0.25	0.16	0.13
	KxS	0.36	0.28	0.28	0.19
	Low	0.24	0.31	0.27	0.21
	N1	0.25	0.35	0.31	0.23
	N1EM	0.25	0.2	0.26	0.19
	S	0.17	0.12	0.12	0.05
IVDMD	All	0.69	0.67	0.67	0.66
	K	0.13	0.15	0.19	0.04
	KxS	0.39	0.27	0.26	0.27
	Low	0.26	0.27	0.29	0.3
	N1	0.14	0.18	0.16	0.24
	N1EM	0.19	0.13	0.19	0.19
	S	0.12	0.02	0.05	0.04
NSC	All	0.6	0.61	0.6	0.57
	K	0.18	0.15	0.1	0.34
	KxS	0.38	0.33	0.31	0.4
	Low	0.23	0.28	0.24	0.3
	N1	0.15	0.25	0.26	0.16
	N1EM	0.13	0.18	0.23	0.27
	S	0.25	0.34	0.29	0.01
NSCE	All	0.58	0.59	0.57	0.61
	K	0.14	0.13	0.04	0.16
	KxS	0.39	0.37	0.34	0.4
	Low	0.24	0.29	0.24	0.21
	N1	0.11	0.22	0.18	0.12
	N1EM	0.17	0.26	0.26	0.19
	S	0.29	0.38	0.29	0.27
PCA	All	0.74	0.73	0.74	0.75
	K	0.15	0.13	0.17	0.12
	KxS	0.38	0.26	0.25	0.22
	Low	0.31	0.29	0.32	0.21
	N1	0.07	0.13	0.08	0.13
	N1EM	0.52	0.47	0.52	0.48
	S	0.36	0.39	0.41	0.2
PPEN	All	0.73	0.73	0.72	0.74
	K	0.3	0.28	0.27	0.44
	KxS	0.35	0.37	0.34	0.16
	Low	0.24	0.27	0.25	0.31
	N1	0.11	0.23	0.18	0.27
	N1EM	0.31	0.25	0.26	0.24
	S	0.23	0.27	0.24	0.16
PSOL	All	0.58	0.58	0.57	0.56
	K	0.14	0.15	0.08	0.05
	KxS	0.42	0.36	0.33	0.42
	Low	0.25	0.28	0.25	0.24
	N1	0.18	0.26	0.24	0.28
	N1EM	0.11	0.16	0.19	0.18
	S	0.24	0.31	0.28	0.21
SC	All	0.66	0.67	0.67	0.64
	K	0.16	0.18	0.11	0.29
	KxS	0.47	0.43	0.42	0.42
	Low	0.3	0.35	0.33	0.34
	N1	0.15	0.25	0.25	0.15
	N1EM	0.15	0.21	0.25	0.31
	S	0.25	0.33	0.3	0.1
SUC	All	0.68	0.68	0.67	0.7
	K	0.13	0.17	0.06	0.14
	KxS	0.49	0.44	0.4	0.42
	Low	0.28	0.3	0.27	0.31
	N1	0.21	0.29	0.28	0.12
	N1EM	0.13	0.14	0.16	−0.1
	S	0.19	0.17	0.14	0.18
UA	All	0.63	0.62	0.62	0.61
	K	0.11	0.18	0.08	0.14
	KxS	0.21	0.21	0.22	0.19
	Low	0.24	0.26	0.27	0.28
	N1	0.03	0.08	0.12	0.34
	N1EM	0.33	0.38	0.41	0.46
	S	0.01	−0.02	0.01	0.09
YLD	All	0.66	0.62	0.65	0.65
	K	0.02	−0.03	0.09	0.2
	KxS	0	−0.12	0.09	−0.03
	Low	0.14	0.11	0.05	0.22
	N1	0	0.07	−0.08	0.16
	N1EM	0.13	0.06	0.02	0.24
	S	0.25	0.15	0.24	0.14

^a^Validation strategies were designed to estimate prediction accuracy within the indicated population by performing 5-fold cross validation with each validation set comprised of 20% of the indicated population

^b^*r* correlation of GEBV with observed phenotypic values, GS accuracy

^c^Prediction accuracies above 0.4 are highlighted. See Additional file 1: Tables S1–4 for mean square error (MSE), intercept, slope, and standard deviation statistics

^d’^“Low” represents the combined N1, N1EM, and K populations

**Fig. 3:**
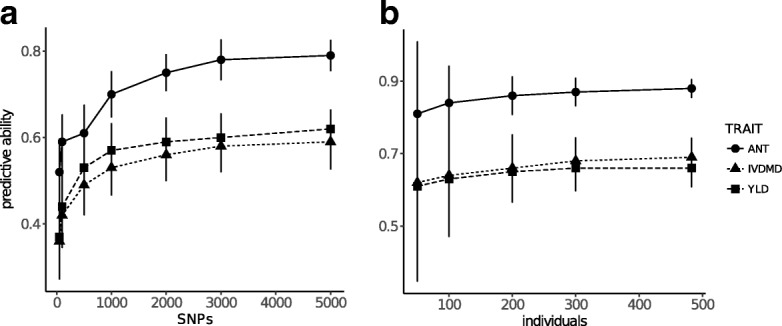
Differing numbers of SNPs or numbers of individuals in the kin-BLUP model. SNPs and individuals were randomly sampled at the indicated levels and then used to construct the realized relationship matrix. Error bars: ± standard deviation of predictive ability (accuracy). **a** Different numbers of SNPs using all available individuals from all populations. **b** Random subsampling of the numbers of individuals using all available SNPs

The influence of increasing SNP density as well as the training population size on prediction accuracy was analyzed as before using kin-BLUP with 5-fold CV across all individuals for three test traits: IVDMD, YLD, and ANT. The results are shown in Fig. 3a and b. For these traits increasing the number of SNPs beyond 50 that were included in the CS improved accuracy 50.0–62.2% with little improvement seen beyond 3000 SNP. The effect of increasing the number of individuals comprising the CS from 40 to 386 resulted in a 7.6–10.1% improvement in prediction accuracy and a decrease in its standard deviation across replicates and folds.

When the CV approach was unconstrained by population different regression techniques produced very similar prediction accuracies for all traits except GRN. For GRN, the SPLS method produced the highest accuracy (*r* = 0.74, MSE = 0.31) while PLS produced the lowest (*r* = 0.65, MSE = 0.34). When the CV strategy was constrained by individual population or within the lowland (“Low”) populations collectively, the results among regression techniques were more variable. The greatest variation among methods occurred for the trait·population combinations where maximum prediction accuracy was below 0.3. However, the coefficient of variation was greater than 20% between methods for some trait·population combinations with accuracies above 0.3. These included the NSCE·S, PPEN·KxS, NSC·S, PCA·Low, GRN·S, and SC·S combinations. In these cases, SPLS or PLS were the most reliable prediction methods, while BB was the least reliable.

Analysis of variance was used to quantify the influences of trait, population, and prediction method on both accuracy and bias of GEBV measurements (Additional file 1: Table S6). GEBV prediction accuracy was heavily influenced by both CV strategy and trait and their second-order interactions with prediction method. The influence of prediction method alone was only suggestive of a significant effect. Bias was also impacted to a large degree by population and trait and their interaction effects while the second-order interaction of trait x prediction method was only suggestive of some influence.

## Discussion

### GBS

Genotyping by sequencing approaches have been used with varying numbers of loci and different genome coverages for GS purposes in many species. Previously in switchgrass, both reference based and non-reference based SNP calling methods have been applied to GBS data and these methods have provided evidence that marker discovery and GS breeding approaches may be both cost-effective and accurate [5, 31, 44]. In this study, the ability to call SNP accurately was facilitated with the TASSEL-GBSv2 pipeline. Numbers of tags and SNPs obtained using this method were comparable to other species such as oil palm (*Elaeis guineensis*; 19,432 SNPs) and wheat (*Triticum aestivum*; 34,749) [10, 43]. The tags and SNPs aligned unevenly across the reference genome with more located toward the ends of each chromosome and with no significant bias toward either sub-genome. Using *PstI* with a recognition sequence of ‘CTGCAT’ to assemble the libraries may have resulted in fewer markers aligned to repetitive elements of low GC content and in more markers associated with the coding or regulatory regions of the reference sequence located in subtelomeric regions. This uneven distribution is difficult to separate from the current state of the available switchgrass reference genome which is fragmentary, but consistent with known gene distribution in other grass species such as sorghum (*Sorghum bicolor*; [42]).

### Population structure

Many of the trait phenotypes and the molecular variation displayed moderate to severe population structure. This was expected given the breeding history and origination of these lines. The lowland populations K, N1, and N1EM were less differentiated among themselves. Large differences in the estimation of genomic heritability occurred depending on population and trait. Except for a few traits (PSOL, NSC, ASH, FAT), most of the heritability existed across populations rather than within populations. This contrasts with some findings in maize (*Zea mays*) and rice (*Oryza sativa*) populations where most heritability could be explained within populations [21].

Accuracy and reliability of genomic predictions, have been shown to decline as the distance between validation and training set increases [26]. In order to avoid this situation, random sampling combined with a large CS enabled capturing most of the existing diversity in the CS across populations. Others have dealt with this issue by stratified sampling across populations to optimize the calibration set or have used criterion such as reliability [57], prediction error variance, or coefficients of determination [1, 26, 50]. These choices are particularly relevant within a breeding program and depend on the interaction of trait architecture and degree of structure present.

### Genomic prediction

The repeatability of measured traits was between 0.34 and 0.94. A strong positive relationship (R^2^ = 0.7; data not shown) exists between repeatability and GEBV accuracy and more focus was given to the 21 traits that had both reasonable repeatability and accuracy above 0.5 for further analysis using different prediction approaches. These traits included total hexoses and ethanol potential, which are both important parameters for bioenergy, as well as IVDMD which is predictive of daily weight gains in cattle. Some traits that resulted in poor GEBV accuracy included CAL, GAL, N, STA, MAN, FUC, GLC and DM. One or more of these traits may have had better accuracy using a restricted VS. However, these traits were not examined due to the poor overall accuracy. Although yield was included in this study, yield data were collected in a spaced-planting trial whic is not an accurate indicator of yield in densely-planted (sward) plots with higher plant competition and mortality rates [5, 44]. This fact complicates genomic selection strategies that include yield, but even including progeny testing in sward plots, GS breeding strategies can still shorten the generation interval. Prediction accuracy for switchgrass yield in swards has been reported to be between 0.145 and 0.237 depending on the genotyping platform [5].

Similar to previous examples of genomic prediction based on GBS [10], reducing the number of SNP markers used in analysis (to approximately 3000 in this case) did not adversely affect predictive ability when the VS was unconstrained among all populations. The accuracy of GEBVs using kin-BLUP increased up to the inclusion of 3000 SNP markers for YLD, ANT, and IVDMD. This could be due to the ability of relatively few markers to adequately capture the relationships between populations, as well as the relatively close relationships of individuals within each population. Higher marker coverage would be necessary to adequately capture more genetic variance associated with QTL in common between populations. Indeed, predictive ability within individual populations was poor in many cases and average inter-marker interval was much greater than the distance threshold for LD (*r*^2^ ≤ 0.2). LD was found in this study to decline rapidly over short distances < 1000 bp. This rapid LD decay could be explained by the outcrossing nature of switchgrass [34], large effective population size, high recombination rates or other mechanisms [22]. These rates were similar to those reported for US inbreds and landraces in *Zea mays* [55] and in *Picea abies* [25]. Increasing the number of individuals included in the kin-BLUP analysis from 50 to 483 led to moderate, linear increases in average prediction accuracy with a lower standard deviation between replicates using 5-fold cross validation.

Differences between prediction methods were of interest as there are very few empirical examples of direct comparisons among these methods using actual data rather than simulated data. The PLS method employs dimensionality reduction while the sparse PLS combines both dimensionality reduction and variable selection. BayesB also uses variable selection as it allows some markers to have large effects and most to have a genetic variance of 0. Previously, results with switchgrass showed no discernible performance differences with three different prediction methods [31]. This study has shown also that the methods were overall very similar in prediction abilities across different traits and VS. Variation between methods was observed relative to trait·population combinations with lower accuracies. These combinations had higher standard deviations between replicates and across folds, so in large part this variation is likely to represent the random error component. However, several trait·population combinations stood out. These included the estimated ethanol from non-structural carbohydrates (NSCE), soluble glucose (GLCS), sucrose (SUC), and non-structural carbohydrates (NSC) particularly in the Summer population where the PLS method performed substantially better than the other methods. It is not surprising in this case that these traits should act similarly as these phenotypes were positively correlated and NSCE and NSC are calculated directly from the NIR estimates of STA, GLCS, FRU, and SUC [60]. Another combination that is interesting are the estimates of p-coumaric esters (PCA) and ester-linked ferulates (FETH). These traits are positively correlated and have the lowest values in the Summer population. The accuracies of the GEBV were relatively high when the VS consisted of any population, except Kanlow and in the specific case of PCA in Kanlow N1. This is only exceptional because, for these two traits, there appears to be no strong reason why there should be such a range of prediction accuracies across the different populations, within the same trait among the lowland populations, given they were so closely related based on PCA and *F* statistics.

The prediction accuracies in this set of data are comparable to those found among morphological and compositional traits in a Northern upland diversity panel [31]. In that study, some of the same traits were analyzed, the CS and VS were derived from all the lines, and the first two principal components of a PCA were used as explanatory variables to account for population structure. In the present study, the first three principle components were considered only for calculating heritability across populations while the populations were treated independently or in combination during the cross-validation step to evaluate model performance without attempting to use the PC as explanatory variables.

## Conclusions

This study emphasizes the possible benefits of using genomic selection in switchgrass. There are still many improvements that can be made in both phenotyping and genotyping methods (such as efficient indirect methods to predict yield in highly competitive environments) to improve genetic gains more rapidly in switchgrass [5]. However, it is apparent that both morphological and compositional traits may be efficiently selected using indirect methods based on genotypic data alone and whole genome regression techniques. The key in the future will be to assess the cost effectiveness of these techniques in switchgrass, given the uncertainty and complexity of bioenergy production processes.

## Additional file

Additional file 1: **Figure S1.** Correllelogram depicting positive (blue) and negative (red) correlations among whole plant traits. Color scale on right indicates Pearson correlation coefficient *r*. **Figure S2.** Correllelogram depicting positive (blue) and negative (red) correlations among wall composition traits determined by NIR. Color scale on right indicates Pearson correlation coefficient *r.* **Figure S3.** Boxplots of (a) ANT, (b) IVDMD, and (c) YLD for each population. Bottom and top of each box represent the first and third quartiles. Horizontal line represents the median, whiskers extend to the most extreme data point that is no more than 1.5 times the interquartile range from the box. **Table S1.** kin-BLUP regression statistics from 20 replicates of 5-fold CV. **Table S2.** Partial Least Squares regression statistics from 20 replicates of 5-fold CV. **Table S3.** Sparse Partial Least Squares Regression statistics from 20 replicates of 5-fold CV. **Table S4.** BayesB Regression statistics from 5-fold CV. Using 5000 iterations and a 1500 iteration burn-in period (see Methods Section). **Table S5.** Variance components for selected traits after partitioning based on dominant principal components 1–3. Table S6: ANOVA of factors influencing prediction accuracy. (DOCX 442 kb)

## Abbreviations

ADF: Acid detergent fiber
ADL: Acid detergent lignin
ANT: anthesis date
ANT: Anthesis Date
ARA: Arabinose
ASH: Minerals (total ash)
AX: ARA + XYL
AXMG: ARA + XYL + Man+GAL
BB: BayesB
BLUP: best linear unbiased predictors
C: Carbon
CAL: Total energy content
CS: calibration set
CTAB: Cetyltrimethylammonium bromide
CV: cross validation
CWC: Cell wall concentration
CWE: Cell wall ethanol
CWEP: Theoretical ethanol conversion efficiency from cell wall hexosans
DM: Dry Matter
DOY: day of year
ETOH: Ethanol/g dry forage
FAT: Extracted fat
FEST: Esterified ferulates
FETH: Etherified ferulates
FRU: Fructose
FUC: Fucose
GAL: Galactose
GBS: genotyping by sequencing
gDNA: genomic DNA
GEBV: genomic breeding values
GLC: Glucose
GLCS: Soluble glucose
GRN: spring emergence date
GRN: Spring Emergence Date
GS: genomic selection
HEX: Total hexoses
HEXE: Theoretical ethanol from hexoses (excluding starch)
HEXEP: Hexose ethanol extraction efficiency
IVDMD: In vitro dry matter digestibility
K: Kanlow
kin-BLUP: kinship best linear unbiased prediction
KL: Klason Lignin
KxS: Kanlow x Summer F2
LD: linkage disequilibrium
Low: lowland
MAN: Mannose
MANOVA: multivariate analysis of variance
MSE: mean squared error
N: Nitrogen
N1: Kanlow N1
N1EM: Kanlow N1-early maturity
NDF: Neutral detergent fiber
NIRS: near infrared spectroscopy
NSC: Non-structural carbohydrates (starch + SC)
NSCE: Estimated ethanol from non-structural carbohydrates
PC: principle component
PCA: p-Coumarate esters
PCA: principle component analysis, p-coumaric acid
PCR: polymerase chain reaction
PENT: Pentose sugars relased/g dry forage
PENTP: Pentoses extraction efficiency
PLS: partial least squares
PPEN: Pentose proportion of total carbohydrates
PSOL: Proportion of hexoses that are non-structural or soluble
QTL: quantitative trait locus
REML: restricted maximum likelihood
RHA: Rhamnose
S: Summer
SC: Total soluble carbohydrates
SNP: single nucleotide polymorphism
SPLS: sparse partial least squares
STA: Starch
SUC: Sucrose
SUG: Total sugars
TsTv: transition:transversion ratio
UA: Uronic Acids
UTR: untranslated region
VS: validation set
XYL: Xylose
YLD: dry weight yield
YLD: Dry matter Yield

## References

[1] Akdemir D, Sanchez JI, Jannink J-L (2015) Optimization of genomic selection training populations with a genetic algorithm. Genet Sel Evol GSE 47:38. https://doi.org/10.1186/s12711-015-0116-6. 10.1186/s12711-015-0116-6 PMC4422310 25943105

[2] Bates D, Mächler M, Bolker BM, Walker SC (2015) Fitting linear mixed-effects models using lme4. J Stat Softw 67:1–48. https://doi.org/10.18637/jss.v067.i01.

[3] Bouton J (2007). The economic benefits of forage improvement in the United States. Euphytica Neth J Plant Breed.

[4] Casler M (2005). Ecotypic variation among switchgrass populations from the northern USA. Crop Sci.

[5] Casler MD, Ramstein GP (2017) Breeding for biomass yield in switchgrass using surrogate measures of yield. BioEnergy Res 1–12. https://doi.org/10.1007/s12155-017-9867-y.

[6] Chen D-H, Ronald P (1999). A rapid DNA miniprepreparation method suitable for AFLP and other PCR application. Plant Mol Biol Rep.

[7] Chun H, Keleş S (2010) Sparse partial least squares regression for simultaneous dimension reduction and variable selection. J R Stat Soc Ser B Stat Methodol 72:3–25. https://doi.org/10.1111/j.1467-9868.2009.00723.x. 10.1111/j.1467-9868.2009.00723.x PMC2810828 20107611

[8] Chung D, Chun H, Keleş S (2013). spls: Sparse Partial Least Squares (SPLS) Regression and Classification.

[9] Cingolani P, Platts A, Wang LL, et al (2012) A program for annotating and predicting the effects of single nucleotide polymorphisms, SnpEff: SNPs in the genome of *Drosophila melanogaster* strain w1118; iso-2; iso-3. Fly (Austin) 6:80–92. https://doi.org/10.4161/fly.19695. 10.4161/fly.19695 PMC3679285 22728672

[10] Cros D, Bocs S, Riou V, et al (2017) Genomic preselection with genotyping-by-sequencing increases performance of commercial oil palm hybrid crosses. BMC Genomics 18:839. https://doi.org/10.1186/s12864-017-4179-3. 10.1186/s12864-017-4179-3 PMC5667528 29096603

[11] De Los Campos G, Rodriguez PP (2016) BGLR: Bayesian generalized linear regression. https://CRAN.R-project.org/package=BGLR.

[12] De Los Campos G, Gianola D, Rosa GJM, Weigel KA, Crossa J (2010) Semi-parametric genomic-enabled prediction of genetic values using reproducing kernel Hilbert spaces methods. Genet Res 92:295–308. https://doi.org/10.1017/S0016672310000285. 10.1017/S0016672310000285 20943010

[13] Elshire RJ, Glaubitz JC, Sun Q, et al (2011) A robust, simple genotyping-by-sequencing (GBS) approach for high diversity species. PLoS One 6:19379. https://doi.org/10.1371/journal.pone.0019379. 10.1371/journal.pone.0019379 PMC3087801 21573248

[14] Endelman JB (2011) Ridge regression and other kernels for genomic selection with R package rrBLUP. Plant Genome, 4:250–255. https://doi.org/10.3835/plantgenome2011.08.0024..

[15] Endelman JB, Jannink J-L (2013) Shrinkage estimation of the realized relationship matrix. G3 2:1405–1413. https://doi.org/10.1534/g3.112.004259. 10.1534/g3.112.004259 PMC3484671 23173092

[16] Evans J, Crisovan E, Barry K, et al (2015) Diversity and population structure of northern switchgrass as revealed through exome capture sequencing. Plant J 84:800–815. https://doi.org/10.1111/tpj.13041. 10.1111/tpj.13041 26426343

[17] Fike J, Parrish D, Wolf D (2006). Switchgrass production for the upper southeastern USA: influence of cultivar and cutting frequency on biomass yields. Biomass Bioenergy.

[18] García-Ruiz A, Cole JB, VanRaden PM, et al (2016) Changes in genetic selection differentials and generation intervals in US Holstein dairy cattle as a result of genomic selection. Proc Natl Acad Sci U S A 113:E3995–E4004. https://doi.org/10.1073/pnas.1519061113. 10.1073/pnas.1519061113 PMC4948329 27354521

[19] Glaubitz JC, Casstevens TM, Lu F, et al (2014) TASSEL-GBS: a high capacity genotyping by sequencing analysis pipeline. PLoS One 9:e90346. https:/doi.org/10.1371/journal.pone.0090346.. 10.1371/journal.pone.0090346 PMC3938676 24587335

[20] Gois IB, Borém A, Cristofani-Yaly M, et al (2016) Genome wide selection in Citrus breeding. Genet Mol Res 15:gmr15048863. https://doi.org/10.4238/gmr15048863. 10.4238/gmr15048863 27813590

[21] Guo Z, Tucker DM, Basten CJ, et al (2014) The impact of population structure on genomic prediction in stratified populations. Theor Appl Genet 127:749–762. https://doi.org/10.1007/s00122-013-2255-x. 10.1007/s00122-013-2255-x 24452438

[22] Gupta PK, Rustgi S, Kulwal PL (2005). Linkage disequilibrium and association studies in higher plants: present status and future prospects. Plant Mol Biol.

[23] Habier D, Tetens J, Seefried FR, Lichtner P, Thaller G (2010). The impact of genetic relationship information on genomic breeding values in German Holstein cattle. Genet Sel Evol.

[24] Heffner EL, Lorenz AJ, Jannink J-L, Sorrells ME (2010) Plant breeding with genomic selection: gain per unit time and cost. Crop Sci 50:1681–1690. https://doi.org/10.2135/cropsci2009.11.0662.

[25] Heuertz M, De Paoli E, Källman T, et al (2006) Multilocus patterns of nucleotide diversity, linkage disequilibrium and demographic history of Norway spruce [*Picea abies* (L.) karst]. Genetics 174:2095–2105. https://doi.org/10.1534/genetics.106.065102. 10.1534/genetics.106.065102 PMC1698656 17057229

[26] Isidro J, Jannink J-L, Akdemir D, et al (2015) Training set optimization under population structure in genomic selection. Theor Appl Genet 128:145–158. https://doi.org/10.1007/s00122-014-2418-4. 10.1007/s00122-014-2418-4 PMC4282691 25367380

[27] Janss L, De Los Campos G, Sheehan N, Sorensen D. (2012) Inferences from genomic models in stratified populations. Genetics 192:693–704. https://doi.org/10.1534/genetics.112.141143. 10.1534/genetics.112.141143 PMC3454890 22813891

[28] Koshi PT, Stubbendieck J, Eck HV, McCully WG (1982). Switchgrasses: forage yield, forage quality and water-use efficiency. J Range Manag.

[29] Lê Cao K-A, Rossouw D, Robert-Granié C, Besse P (2008) A sparse PLS for variable selection when integrating omics data. Stat Appl Genet Mol Biol 7:Article 35. https://doi.org/10.2202/1544-6115.1390. 10.2202/1544-6115.1390 19049491

[30] Li H, Durbin R (2010) Fast and accurate long-read alignment with burrows-wheeler transform. Bioinforma Oxf Engl 26:589–595. https://doi.org/10.1093/bioinformatics/btp698. 10.1093/bioinformatics/btp698 PMC2828108 20080505

[31] Lipka AE, Lu F, Cherney JH, et al (2014) Accelerating the switchgrass (*Panicum virgatum* L.) breeding cycle using genomic selection approaches. PLoS One 9:e112227. https://doi.org/10.1371/journal.pone.0112227. 10.1371/journal.pone.0112227 PMC4229143 25390940

[32] Makowsky R, Pajewski NM, Klimentidis YC, et al (2011) Beyond missing heritability: prediction of complex traits. PLoS Genet 7:e1002051. https://doi.org/10.1371/journal.pgen.1002051. 10.1371/journal.pgen.1002051 PMC3084207 21552331

[33] Mardia KV, Kent J, Bibby J (1979). Multivariate Analysis.

[34] Martínez-Reyna J, Vogel K (2002). Incompatibility systems in switchgrass. Crop Sci.

[35] Meuwissen T, Hayes B, Goddard M (2001). Prediction of total genetic value using genome-wide dense marker maps. Genetics.

[36] Mevik B-H, Wehrens R (2007). The pls package: principal component and partial least squares regression in R. J Stat Softw.

[37] Missaoui A, Paterson A, Bouton J (2006) Molecular markers for the classification of switchgrass (*Panicum virgatum* L.) germplasm and to assess genetic diversity in three synthetic switchgrass populations. Genet Resour Crop Evol 53:1291–1302. https://doi.org/10.1007/s10722-005-3878-9.

[38] Money D, Gardner K, Migicovsky Z, Schwaninger H, Zhong G, Myles S (2015). LinkImpute: fast and accurate genotype imputation for nonmodel organisms. G3: Genes, Genomes, Genetics.

[39] Moore KJ, Moser LE, Vogel KP, Waller SS, Johnson BE, Pedersen JF (1991). Describing and quantifying growth stages of perennial forage grasses. Agron J.

[40] Okada M, Lanzatella C, Tobias CM (2010) Single-locus EST-SSR markers for characterization of population genetic diversity and structure across ploidy levels in switchgrass (*Panicum virgatum* L.). Genet Resour Crop Evol 58:919–931. https://doi.org/10.1007/s10722-010-9631-z.

[41] Parrish D, Fike J (2005). The biology and agronomy of switchgrass for biofuels. Crit Rev Plant Sci.

[42] Paterson AH, Bowers JE, Bruggmann R (2009). The *Sorghum bicolor* genome and the diversification of grasses. Nature.

[43] Poland JA, Brown PJ, Sorrells ME, Jannink J-L (2012) Development of high-density genetic maps for barley and wheat using a novel two-enzyme genotyping-by-sequencing approach. PLoS One 7:e32253. https://doi.org/10.1371/journal.pone.0032253. 10.1371/journal.pone.0032253 PMC3289635 22389690

[44] Price DL, Casler MD (2014) Divergent selection for secondary traits in upland tetraploid switchgrass and effects on sward biomass yield. BioEnergy Res 7:329–337. https://doi.org/10.1007/s12155-013-9374-8.

[45] Price AL, Zaitlen NA, Reich D, Patterson N (2010) New approaches to population stratification in genome-wide association studies. Nat Rev Genet 11:459–463. https://doi.org/10.1038/nrg2813. 10.1038/nrg2813 PMC2975875 20548291

[46] R Development Core Team. R: A language and environment for statistical computing. r foundation for statistical computing. Vienna, Austria; 2017: https://www.R-project.org.

[47] Ramstein GP, Evans J, Kaeppler SM, et al (2016) Accuracy of genomic prediction in switchgrass (*Panicum virgatum* L.) improved by accounting for linkage disequilibrium. G3 Bethesda Md 6:1049–1062. https://doi.org/10.1534/g3.115.024950. 10.1534/g3.115.024950 PMC4825640 26869619

[48] Resende MFR, Muñoz P, Acosta JJ, et al (2012) Accelerating the domestication of trees using genomic selection: accuracy of prediction models across ages and environments. New Phytol 193:617–624. https://doi.org/10.1111/j.1469-8137.2011.03895.x. 10.1111/j.1469-8137.2011.03895.x 21973055

[49] Riedelsheimer C, Czedik-Eysenberg A, Grieder C, et al (2012) Genomic and metabolic prediction of complex heterotic traits in hybrid maize. Nat Genet 44:217–220. https://doi.org/10.1038/ng.1033. 10.1038/ng.1033 22246502

[50] Rincent R, Laloë D, Nicolas S, et al (2012) Maximizing the reliability of genomic selection by optimizing the calibration set of reference individuals: comparison of methods in two diverse groups of maize inbreds (*Zea mays* L.). Genetics 192:715–728. https://doi.org/10.1534/genetics.112.141473. 10.1534/genetics.112.141473 PMC3454892 22865733

[51] Schmer M, Vogel K, Mitchell R, Perrin R (2008). Net energy of cellulosic ethanol from switchgrass. Proc Natl Acad Sci U S A.

[52] Simeão Resende RM, Casler MD, Vilela de Resende MD (2014) Genomic selection in forage breeding: accuracy and methods. Crop Sci 54:143. https://doi.org/10.2135/cropsci2013.05.0353.

[53] Talbert LE, Timothy DH, Burns JC (1983). Estimates of genetic parameters in switchgrass^1^. Crop Sci.

[54] Taliaferro C (2002). Breeding and selection of new switchgrass varieties for increased biomass production. ORNLSUB-02-19XSY162C01.

[55] Tenaillon MI, Sawkins MC, Long AD, Gaut RL, Doebley JF, Gaut BS (2001). Patterns of DNA sequence polymorphism along chromosome 1 of maize (*Zea mays* ssp. *mays* L.). Proc Natl Acad Sci U S A.

[56] Vallejo RL, Leeds TD, Gao G, et al (2017) Genomic selection models double the accuracy of predicted breeding values for bacterial cold water disease resistance compared to a traditional pedigree-based model in rainbow trout aquaculture. Genet Sel Evol 49:17. https://doi.org/10.1186/s12711-017-0293-6. 10.1186/s12711-017-0293-6 PMC5289005 28148220

[57] VanRaden P (2008). Efficient methods to compute genomic predictions. J Dairy Sci.

[58] Visscher PM, Yang J, Goddard ME (2010) A commentary on ‘common SNPs explain a large proportion of the heritability for human height’ by Yang et al. (2010). Twin Res Hum Genet 13:517–524. https://doi.org/10.1375/twin.13.6.517. 10.1375/twin.13.6.517 21142928

[59] Vogel KP, Mitchell RB (2008) Heterosis in switchgrass: biomass yield in swards. Crop Sci 48:2159. https://doi.org/10.2135/cropsci2008.02.0117.

[60] Vogel KP, Dien BS, Jung HG, Casler MD, Materson SD (2010). Mitchell RB. Quantifying actual and theoretical ethanol yields for switchgrass strains using NIRS analyses Bioenerg Res.

[61] Vogel KP, Dien BS, Jung HG, et al (2011) Quantifying actual and theoretical ethanol yields for switchgrass strains using NIRS analyses. BioEnergy Res 4:96–110. https://doi.org/10.1007/s12155-010-9104-4.

[62] Weir B, Cockerham C (1984) Estimating F-statistics for the analysis of population structure. Evol 38:1358–1370. https://doi.org/10.2307/2408641. 10.1111/j.1558-5646.1984.tb05657.x 28563791

[63] Wold H (1966). Estimation of principal components and related models by interative least squares.

[64] Wong CK, Bernardo R (2008) Genomewide selection in oil palm: increasing selection gain per unit time and cost with small populations. Theor Appl Genet 116:815–824. https://doi.org/10.1007/s00122-008-0715-5. 10.1007/s00122-008-0715-5 18219476

[65] Wray NR, Yang J, Hayes BJ, et al (2013) Pitfalls of predicting complex traits from SNPs. Nat Rev Genet 14:507–515. https://doi.org/10.1038/nrg3457. 10.1038/nrg3457 PMC4096801 23774735

[66] Yu J, Buckler ES (2006). Genetic association mapping and genome organization of maize. Curr Opin Biotechnol.

