# Supplementary material for: Genomic prediction accuracy for switchgrass traits related to bioenergy within differentiated populations
Source: BMC Plant Biol. 2018 Jul 9;18:142. doi: 10.1186/s12870-018-1360-z (PMC6038187; doi:10.1186/s12870-018-1360-z)
Supplement: Supplementary file 1 — Figure S1. Correllelogram depicting positive (blue) and negative (red) correlations among whole plant traits. Color scale on right indicates Pearson correlation coefficient r. Figure S2. Correllelogram depicting positive (blue) and negative (red) correlations among wall composition traits determined by NIR. Color scale on right indicates Pearson correlation coefficient r. Figure S3. Boxplots of (a) ANT, (b) IVDMD, and (c) YLD for each population. Bottom and top of each box represent the first and third quartiles. Horizontal line represents the median, whiskers extend to the most extreme data point that is no more than 1.5 times the interquartile range from the box. Table S1. kin-BLUP regression statistics from 20 replicates of 5-fold CV. Table S2. Partial Least Squares regression statistics from 20 replicates of 5-fold CV. Table S3. Sparse Partial Least Squares Regression statistics from 20 replicates of 5-fold CV. Table S4. BayesB Regression statistics from 5-fold CV. Using 5000 iterations and a 1500 iteration burn-in period (see Methods Section). Table S5. Variance components for selected traits after partitioning based on dominant principal components 1–3. Table S6: ANOVA of factors influencing prediction accuracy. (DOCX 442 kb) [file 12870_2018_1360_MOESM1_ESM.docx]

Supplemental Data: Fiedler et al. Genomic Prediction Accuracy for Switchgrass Traits Related to Bioenergy Within Differentiated Populations


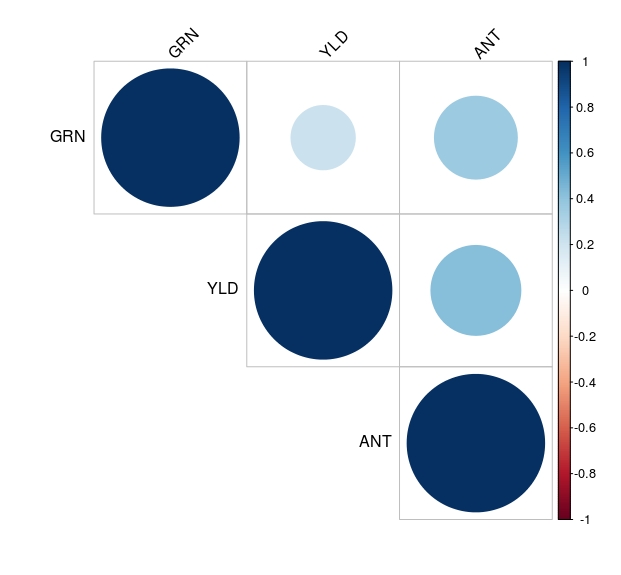


Figure S1. Correllelogram depicting positive (blue) and negative (red) correlations among whole plant traits. Color scale on right indicates Pearson correlation coefficient *r*.


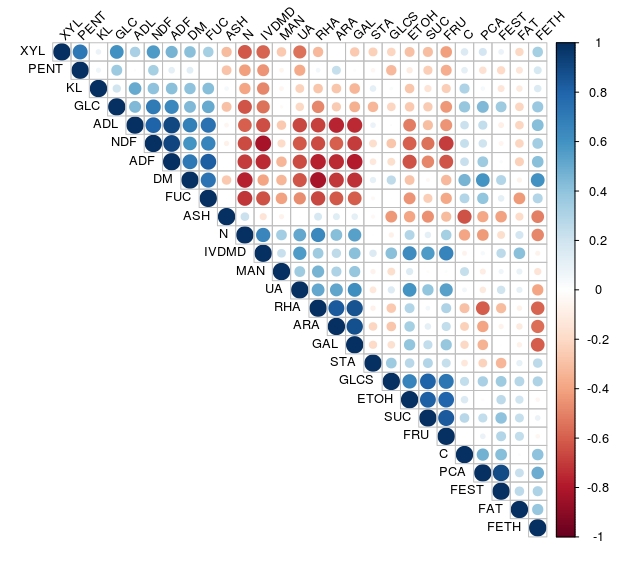


Figure S2. Correllelogram depicting positive (blue) and negative (red) correlations among wall composition traits determined by NIR. Color scale on right indicates Pearson correlation coefficient *r.*


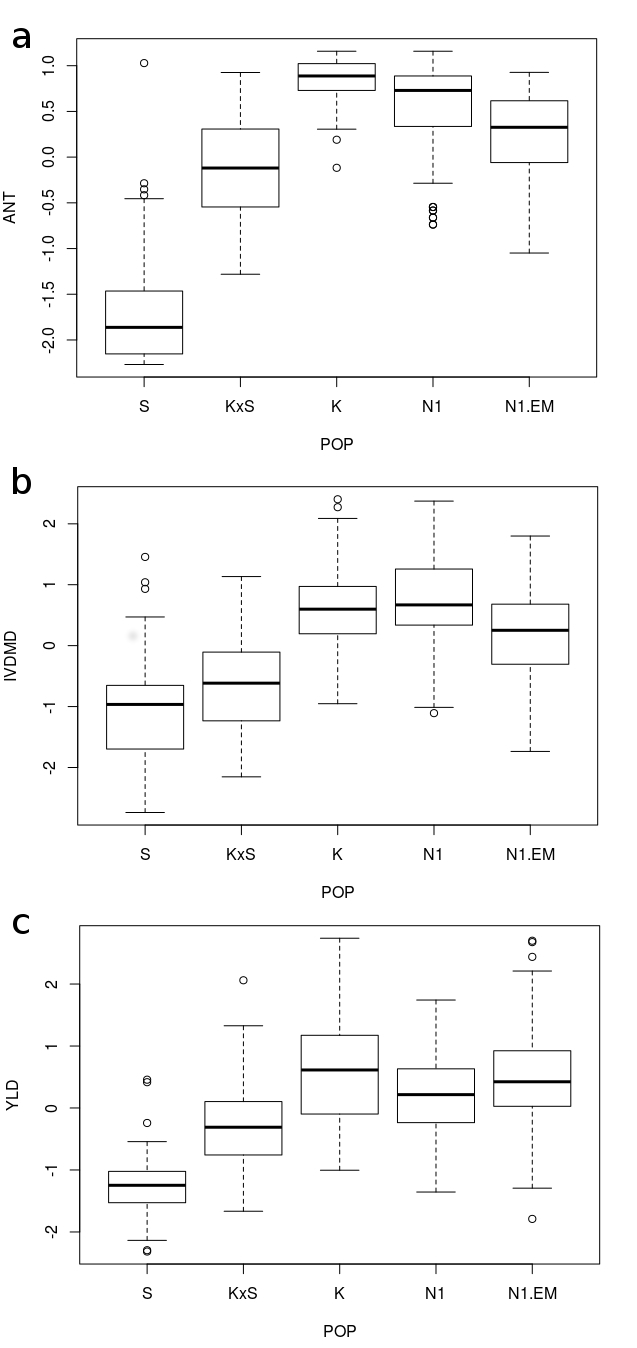


Figure S3. Boxplots of (a) ANT, (b) IVDMD, and (c) YLD for each population. Bottom and top of each box represent the first and third quartiles. Horizontal line represents the median, whiskers extend to the most extreme data point that is no more than 1.5 times the interquartile range from the box.

| Table S1: kin-BLUP regression statistics from 20 replicates of 5-fold CV. | | | | | |
| --- | --- | --- | --- | --- | --- |
| **TRAIT** | **POP** | **MSE^1^** | **INTER^2^** | **SLOPE** | **STDEV^3^** |
| ANT | All | 0.18 | 0 | 0.95 | 0.026 |
|  | K | 0.06 | 0.69 | 0.09 | 0.197 |
|  | KxS | 0.25 | -0.03 | 0.54 | 0.216 |
|  | Low | 0.12 | -0.12 | 1.18 | 0.07 |
|  | N1 | 0.16 | -0.16 | 1.27 | 0.158 |
|  | N1EM | 0.16 | -0.03 | 0.73 | 0.195 |
|  | S | 0.31 | -1.28 | 0.16 | 0.341 |
| ASH | All | 0.19 | 0 | 0.72 | 0.061 |
|  | K | 0.14 | -0.02 | 0.52 | 0.215 |
|  | KxS | 0.2 | 0.09 | 0.71 | 0.186 |
|  | Low | 0.13 | -0.05 | 0.56 | 0.11 |
|  | N1 | 0.12 | -0.09 | 0.47 | 0.213 |
|  | N1EM | 0.13 | -0.14 | 0.43 | 0.216 |
|  | S | 0.39 | -0.04 | 0.7 | 0.183 |
| AX | All | 0.24 | 0 | 0.7 | 0.066 |
|  | K | 0.19 | -0.15 | 0.41 | 0.217 |
|  | KxS | 0.21 | -0.01 | 0.74 | 0.182 |
|  | Low | 0.23 | -0.07 | 0.51 | 0.092 |
|  | N1 | 0.28 | -0.09 | 0.42 | 0.203 |
|  | N1EM | 0.22 | -0.07 | 0.43 | 0.197 |
|  | S | 0.29 | 0.39 | 0.3 | 0.252 |
| ETOH | All | 0.29 | 0 | 0.82 | 0.059 |
|  | K | 0.25 | 0.02 | 0.64 | 0.209 |
|  | KxS | 0.28 | 0.07 | 1.15 | 0.165 |
|  | Low | 0.28 | 0.03 | 0.76 | 0.109 |
|  | N1 | 0.33 | 0.1 | 0.87 | 0.202 |
|  | N1EM | 0.25 | -0.03 | 0.75 | 0.21 |
|  | S | 0.37 | -0.18 | 0.57 | 0.2 |
| FAT | All | 0.12 | 0 | 0.73 | 0.047 |
|  | K | 0.1 | -0.02 | 0.69 | 0.2 |
|  | KxS | 0.16 | -0.29 | 0.27 | 0.209 |
|  | Low | 0.09 | -0.08 | 1 | 0.096 |
|  | N1 | 0.08 | -0.14 | 1.24 | 0.147 |
|  | N1EM | 0.09 | -0.09 | 0.99 | 0.172 |
|  | S | 0.19 | -0.35 | -0.02 | 0.225 |
| FEST | All | 0.21 | 0 | 0.9 | 0.028 |
|  | K | 0.14 | 0.39 | 0.28 | 0.232 |
|  | KxS | 0.28 | 0.08 | 1.13 | 0.163 |
|  | Low | 0.17 | -0.03 | 0.96 | 0.091 |
|  | N1 | 0.2 | 0.04 | 0.82 | 0.231 |
|  | N1EM | 0.17 | -0.18 | 1.17 | 0.182 |
|  | S | 0.27 | -0.25 | 0.73 | 0.264 |
| FRU | All | 0.25 | 0 | 0.73 | 0.064 |
|  | K | 0.29 | 0.13 | 0.23 | 0.231 |
|  | KxS | 0.15 | 0.12 | 1.3 | 0.149 |
|  | Low | 0.28 | 0.05 | 0.6 | 0.111 |
|  | N1 | 0.3 | 0.26 | 0.46 | 0.172 |
|  | N1EM | 0.24 | 0.04 | 0.17 | 0.21 |
|  | S | 0.26 | -0.38 | 0.23 | 0.265 |
| GLCS | All | 0.29 | 0 | 0.78 | 0.052 |
|  | K | 0.32 | 0.18 | 0.24 | 0.224 |
|  | KxS | 0.25 | 0.08 | 1.17 | 0.135 |
|  | Low | 0.3 | 0.18 | 0.33 | 0.107 |
|  | N1 | 0.3 | 0.31 | 0.33 | 0.21 |
|  | N1EM | 0.27 | 0.18 | 0.08 | 0.206 |
|  | S | 0.35 | -0.2 | 0.55 | 0.225 |
| GRN | All | 0.51 | 0 | 0.43 | 0.271 |
|  | K | 1.45 | 1.36 | -1.85 | 0.238 |
|  | KxS | 0.15 | -0.28 | 0.03 | 0.231 |
|  | Low | 0.6 | 0.48 | -0.38 | 0.141 |
|  | N1 | 0.11 | 0.07 | 0.43 | 0.21 |
|  | N1EM | 0.12 | 0.25 | 0 | 0.219 |
|  | S | 0.7 | -0.35 | 0.16 | 0.346 |
| HEX | All | 0.19 | 0 | 0.81 | 0.038 |
|  | K | 0.19 | -0.18 | 1.24 | 0.194 |
|  | KxS | 0.2 | 0.08 | 1.14 | 0.208 |
|  | Low | 0.16 | 0.08 | 0.64 | 0.1 |
|  | N1 | 0.17 | 0.33 | -0.05 | 0.198 |
|  | N1EM | 0.12 | 0.2 | 0.5 | 0.191 |
|  | S | 0.29 | 0.14 | 0.9 | 0.19 |
| HEXE | All | 0.18 | 0 | 0.7 | 0.056 |
|  | K | 0.14 | -0.13 | 1.06 | 0.185 |
|  | KxS | 0.18 | 0.11 | 1.17 | 0.22 |
|  | Low | 0.14 | 0.03 | 0.6 | 0.089 |
|  | N1 | 0.14 | 0.27 | -0.11 | 0.19 |
|  | N1EM | 0.13 | 0.07 | 0.61 | 0.18 |
|  | S | 0.35 | -0.04 | 0.64 | 0.24 |
| HEXEP | All | 0.19 | 0 | 0.68 | 0.053 |
|  | K | 0.17 | 0.04 | 0.4 | 0.196 |
|  | KxS | 0.17 | 0.06 | 1.01 | 0.161 |
|  | Low | 0.18 | -0.05 | 0.85 | 0.106 |
|  | N1 | 0.18 | 0.08 | 0.86 | 0.198 |
|  | N1EM | 0.19 | -0.12 | 0.82 | 0.207 |
|  | S | 0.28 | -0.09 | 0.53 | 0.195 |
| IVDMD | All | 0.17 | 0 | 0.71 | 0.054 |
|  | K | 0.14 | 0.12 | 0.44 | 0.197 |
|  | KxS | 0.14 | 0.05 | 0.96 | 0.2 |
|  | Low | 0.15 | -0.03 | 0.81 | 0.11 |
|  | N1 | 0.14 | 0.21 | 0.45 | 0.211 |
|  | N1EM | 0.15 | -0.06 | 0.62 | 0.215 |
|  | S | 0.28 | -0.15 | 0.48 | 0.213 |
| NSC | All | 0.33 | 0 | 0.81 | 0.06 |
|  | K | 0.37 | 0.09 | 0.6 | 0.212 |
|  | KxS | 0.31 | 0.02 | 1.05 | 0.175 |
|  | Low | 0.34 | 0.05 | 0.68 | 0.094 |
|  | N1 | 0.39 | 0.24 | 0.52 | 0.208 |
|  | N1EM | 0.26 | 0.02 | 0.32 | 0.193 |
|  | S | 0.32 | -0.05 | 0.69 | 0.201 |
| NSCE | All | 0.3 | 0 | 0.77 | 0.061 |
|  | K | 0.32 | 0.1 | 0.5 | 0.173 |
|  | KxS | 0.29 | 0.04 | 1.06 | 0.164 |
|  | Low | 0.29 | 0.03 | 0.69 | 0.097 |
|  | N1 | 0.33 | 0.25 | 0.43 | 0.213 |
|  | N1EM | 0.23 | -0.03 | 0.39 | 0.202 |
|  | S | 0.3 | 0.16 | 0.91 | 0.203 |
| PCA | All | 0.22 | 0 | 0.82 | 0.039 |
|  | K | 0.16 | 0.17 | 0.58 | 0.25 |
|  | KxS | 0.25 | 0.07 | 1.05 | 0.197 |
|  | Low | 0.18 | -0.1 | 1.05 | 0.106 |
|  | N1 | 0.2 | 0.14 | 0.32 | 0.205 |
|  | N1EM | 0.17 | -0.24 | 1.38 | 0.154 |
|  | S | 0.33 | -0.06 | 0.77 | 0.272 |
| PPEN | All | 0.26 | 0 | 0.83 | 0.041 |
|  | K | 0.25 | 0.15 | 1.26 | 0.192 |
|  | KxS | 0.21 | 0.01 | 0.93 | 0.199 |
|  | Low | 0.25 | 0 | 0.86 | 0.105 |
|  | N1 | 0.32 | -0.11 | 0.41 | 0.168 |
|  | N1EM | 0.18 | -0.03 | 0.83 | 0.193 |
|  | S | 0.3 | 0.23 | 0.61 | 0.227 |
| PSOL | All | 0.32 | 0 | 0.8 | 0.057 |
|  | K | 0.38 | 0.09 | 0.48 | 0.172 |
|  | KxS | 0.27 | 0.04 | 1.1 | 0.157 |
|  | Low | 0.35 | 0.04 | 0.71 | 0.089 |
|  | N1 | 0.41 | 0.22 | 0.62 | 0.216 |
|  | N1EM | 0.26 | -0.01 | 0.24 | 0.222 |
|  | S | 0.27 | -0.07 | 0.67 | 0.211 |
| SC | All | 0.31 | 0 | 0.85 | 0.054 |
|  | K | 0.36 | 0.15 | 0.54 | 0.169 |
|  | KxS | 0.26 | 0.07 | 1.21 | 0.156 |
|  | Low | 0.34 | 0 | 0.85 | 0.094 |
|  | N1 | 0.37 | 0.31 | 0.51 | 0.21 |
|  | N1EM | 0.29 | -0.03 | 0.35 | 0.235 |
|  | S | 0.29 | -0.15 | 0.63 | 0.239 |
| SUC | All | 0.27 | 0 | 0.81 | 0.056 |
|  | K | 0.32 | 0.13 | 0.47 | 0.2 |
|  | KxS | 0.22 | 0.12 | 1.26 | 0.164 |
|  | Low | 0.28 | 0 | 0.84 | 0.104 |
|  | N1 | 0.31 | 0.17 | 0.75 | 0.196 |
|  | N1EM | 0.22 | 0.02 | 0.32 | 0.178 |
|  | S | 0.28 | -0.35 | 0.41 | 0.251 |
| UA | All | 0.16 | 0 | 0.65 | 0.06 |
|  | K | 0.14 | 0.02 | 0.38 | 0.213 |
|  | KxS | 0.14 | -0.06 | 0.49 | 0.252 |
|  | Low | 0.13 | -0.03 | 0.78 | 0.11 |
|  | N1 | 0.12 | 0.22 | 0.12 | 0.232 |
|  | N1EM | 0.15 | -0.09 | 0.96 | 0.176 |
|  | S | 0.28 | -0.42 | 0.12 | 0.249 |
| YLD | All | 0.15 | 0 | 0.58 | 0.053 |
|  | K | 0.17 | 0.22 | 0.17 | 0.18 |
|  | KxS | 0.12 | -0.11 | 0.05 | 0.251 |
|  | Low | 0.15 | -0.01 | 0.62 | 0.107 |
|  | N1 | 0.15 | 0.06 | 0.07 | 0.203 |
|  | N1EM | 0.15 | 0.04 | 0.57 | 0.18 |
|  | S | 0.19 | -0.2 | 0.37 | 0.281 |
| ^1^mean squared error  ^2^average intercept of the regression  ^3^standard deviation of the accuracy across folds and replicates | | | | | |

| Table S2: Partial Least Squares regression statistics from 20 replicates of 5-fold CV. | | | | | |
| --- | --- | --- | --- | --- | --- |
| **TRAIT** | **POP** | **MSE^1^** | **INTER^2^** | **SLOPE** | **SDEV^3^** |
| ANT | All | 0.24 | 0 | 1 | 0.024 |
|  | K | 0.09 | 0.92 | -0.09 | 0.213 |
|  | KxS | 0.34 | -0.05 | 0.5 | 0.191 |
|  | Low | 0.16 | 0.03 | 0.98 | 0.073 |
|  | N1 | 0.21 | -0.06 | 1.14 | 0.161 |
|  | N1EM | 0.21 | 0.09 | 0.45 | 0.192 |
|  | S | 0.41 | -1.47 | 0.15 | 0.341 |
| ASH | All | 0.67 | 0.01 | 0.95 | 0.063 |
|  | K | 0.51 | -0.18 | 0.35 | 0.271 |
|  | KxS | 0.77 | 0.35 | 0.64 | 0.21 |
|  | Low | 0.47 | -0.21 | 0.53 | 0.105 |
|  | N1 | 0.43 | -0.24 | 0.45 | 0.165 |
|  | N1EM | 0.49 | -0.33 | 0.5 | 0.227 |
|  | S | 1.27 | 0.17 | 0.74 | 0.215 |
| AX | All | 0.69 | 0 | 0.89 | 0.058 |
|  | K | 0.57 | -0.38 | 0.28 | 0.214 |
|  | KxS | 0.67 | 0.07 | 0.64 | 0.202 |
|  | Low | 0.68 | -0.24 | 0.41 | 0.103 |
|  | N1 | 0.8 | -0.14 | 0.58 | 0.167 |
|  | N1EM | 0.7 | -0.26 | 0.22 | 0.205 |
|  | S | 0.73 | 0.71 | 0.38 | 0.237 |
| ETOH | All | 0.6 | 0 | 0.97 | 0.053 |
|  | K | 0.48 | 0.03 | 0.8 | 0.147 |
|  | KxS | 0.6 | -0.07 | 0.96 | 0.19 |
|  | Low | 0.57 | 0.1 | 0.8 | 0.116 |
|  | N1 | 0.7 | 0.19 | 0.96 | 0.229 |
|  | N1EM | 0.53 | 0.12 | 0.51 | 0.161 |
|  | S | 0.69 | -0.32 | 0.67 | 0.247 |
| FAT | All | 0.54 | -0.01 | 0.98 | 0.044 |
|  | K | 0.45 | 0.16 | 0.53 | 0.228 |
|  | KxS | 0.82 | -0.81 | 0.17 | 0.177 |
|  | Low | 0.41 | 0.04 | 0.97 | 0.088 |
|  | N1 | 0.36 | -0.18 | 1.47 | 0.145 |
|  | N1EM | 0.39 | 0.04 | 0.94 | 0.184 |
|  | S | 0.71 | -0.75 | 0.03 | 0.236 |
| FEST | All | 0.32 | 0 | 1 | 0.028 |
|  | K | 0.24 | 0.59 | 0.15 | 0.216 |
|  | KxS | 0.46 | -0.02 | 0.96 | 0.214 |
|  | Low | 0.26 | 0.13 | 0.8 | 0.111 |
|  | N1 | 0.29 | 0.07 | 0.88 | 0.207 |
|  | N1EM | 0.25 | -0.01 | 0.93 | 0.197 |
|  | S | 0.36 | -0.03 | 1 | 0.196 |
| FRU | All | 0.66 | 0 | 0.93 | 0.06 |
|  | K | 0.77 | 0.24 | 0.27 | 0.212 |
|  | KxS | 0.48 | -0.05 | 1 | 0.184 |
|  | Low | 0.75 | 0.18 | 0.6 | 0.111 |
|  | N1 | 0.86 | 0.38 | 0.68 | 0.196 |
|  | N1EM | 0.66 | 0.12 | 0.11 | 0.22 |
|  | S | 0.54 | -0.64 | 0.33 | 0.244 |
| GLCS | All | 0.6 | 0 | 0.94 | 0.056 |
|  | K | 0.69 | 0.33 | 0.15 | 0.214 |
|  | KxS | 0.54 | -0.07 | 0.94 | 0.169 |
|  | Low | 0.61 | 0.26 | 0.43 | 0.107 |
|  | N1 | 0.66 | 0.47 | 0.36 | 0.208 |
|  | N1EM | 0.54 | 0.2 | 0.26 | 0.182 |
|  | S | 0.6 | 0.09 | 1.06 | 0.19 |
| GRN | All | 0.83 | 0 | 0.78 | 0.232 |
|  | K | 3.12 | 2.16 | -3.85 | 0.26 |
|  | KxS | 0.17 | -0.59 | -0.28 | 0.183 |
|  | Low | 1.27 | 0.88 | -1.09 | 0.124 |
|  | N1 | 0.21 | 0.52 | -0.06 | 0.241 |
|  | N1EM | 0.14 | 0.4 | -0.04 | 0.239 |
|  | S | 0.16 | -0.7 | 0.21 | 0.314 |
| HEX | All | 0.39 | 0 | 0.98 | 0.039 |
|  | K | 0.42 | 0.24 | 0.58 | 0.208 |
|  | KxS | 0.42 | -0.02 | 1.04 | 0.166 |
|  | Low | 0.35 | 0.28 | 0.5 | 0.095 |
|  | N1 | 0.34 | 0.34 | 0.25 | 0.213 |
|  | N1EM | 0.27 | 0.43 | 0.4 | 0.244 |
|  | S | 0.51 | 0 | 1 | 0.208 |
| HEXE | All | 0.58 | 0 | 0.97 | 0.059 |
|  | K | 0.46 | 0.1 | 0.65 | 0.198 |
|  | KxS | 0.59 | -0.02 | 1.08 | 0.203 |
|  | Low | 0.45 | 0.2 | 0.55 | 0.106 |
|  | N1 | 0.46 | 0.38 | 0.14 | 0.199 |
|  | N1EM | 0.43 | 0.26 | 0.6 | 0.213 |
|  | S | 0.95 | -0.1 | 0.9 | 0.19 |
| HEXEP | All | 0.66 | 0 | 0.95 | 0.061 |
|  | K | 0.55 | 0.05 | 0.63 | 0.161 |
|  | KxS | 0.69 | -0.1 | 0.73 | 0.193 |
|  | Low | 0.63 | 0.06 | 0.87 | 0.106 |
|  | N1 | 0.69 | 0.21 | 1.06 | 0.19 |
|  | N1EM | 0.65 | -0.01 | 0.59 | 0.179 |
|  | S | 0.78 | -0.66 | 0.35 | 0.196 |
| IVDMD | All | 0.56 | -0.01 | 0.95 | 0.05 |
|  | K | 0.46 | 0.38 | 0.36 | 0.162 |
|  | KxS | 0.56 | -0.28 | 0.59 | 0.205 |
|  | Low | 0.5 | 0.19 | 0.66 | 0.089 |
|  | N1 | 0.54 | 0.45 | 0.49 | 0.224 |
|  | N1EM | 0.5 | 0.09 | 0.36 | 0.212 |
|  | S | 0.76 | -0.99 | 0.08 | 0.192 |
| NSC | All | 0.63 | 0 | 0.96 | 0.059 |
|  | K | 0.75 | 0.26 | 0.46 | 0.184 |
|  | KxS | 0.68 | -0.18 | 0.75 | 0.195 |
|  | Low | 0.65 | 0.12 | 0.76 | 0.098 |
|  | N1 | 0.75 | 0.21 | 0.83 | 0.225 |
|  | N1EM | 0.48 | 0.05 | 0.39 | 0.211 |
|  | S | 0.53 | 0.09 | 1.05 | 0.185 |
| NSCE | All | 0.66 | -0.01 | 0.95 | 0.057 |
|  | K | 0.75 | 0.28 | 0.37 | 0.209 |
|  | KxS | 0.72 | -0.13 | 0.83 | 0.176 |
|  | Low | 0.67 | 0.1 | 0.76 | 0.091 |
|  | N1 | 0.77 | 0.29 | 0.71 | 0.2 |
|  | N1EM | 0.49 | -0.03 | 0.57 | 0.177 |
|  | S | 0.55 | 0.19 | 1.15 | 0.203 |
| PCA | All | 0.47 | 0 | 0.99 | 0.043 |
|  | K | 0.39 | 0.44 | 0.38 | 0.19 |
|  | KxS | 0.58 | -0.05 | 0.64 | 0.208 |
|  | Low | 0.39 | 0.12 | 0.74 | 0.1 |
|  | N1 | 0.41 | 0.2 | 0.41 | 0.222 |
|  | N1EM | 0.35 | -0.07 | 1.06 | 0.173 |
|  | S | 0.65 | -0.12 | 0.95 | 0.226 |
| PPEN | All | 0.47 | 0.01 | 0.98 | 0.045 |
|  | K | 0.5 | -0.18 | 0.82 | 0.167 |
|  | KxS | 0.41 | 0.15 | 0.77 | 0.166 |
|  | Low | 0.47 | -0.14 | 0.76 | 0.096 |
|  | N1 | 0.56 | -0.04 | 0.72 | 0.185 |
|  | N1EM | 0.38 | -0.29 | 0.53 | 0.208 |
|  | S | 0.5 | 0.42 | 0.68 | 0.243 |
| PSOL | All | 0.67 | 0 | 0.94 | 0.067 |
|  | K | 0.82 | 0.23 | 0.44 | 0.202 |
|  | KxS | 0.64 | -0.11 | 0.81 | 0.175 |
|  | Low | 0.74 | 0.11 | 0.75 | 0.098 |
|  | N1 | 0.87 | 0.24 | 0.87 | 0.198 |
|  | N1EM | 0.54 | 0 | 0.31 | 0.167 |
|  | S | 0.49 | -0.08 | 0.89 | 0.208 |
| SC | All | 0.55 | -0.01 | 0.98 | 0.044 |
|  | K | 0.66 | 0.24 | 0.56 | 0.194 |
|  | KxS | 0.49 | -0.09 | 0.95 | 0.137 |
|  | Low | 0.59 | 0.07 | 0.88 | 0.09 |
|  | N1 | 0.66 | 0.3 | 0.77 | 0.184 |
|  | N1EM | 0.47 | -0.02 | 0.46 | 0.206 |
|  | S | 0.44 | -0.09 | 0.88 | 0.234 |
| SUC | All | 0.54 | 0 | 0.97 | 0.048 |
|  | K | 0.66 | 0.24 | 0.49 | 0.17 |
|  | KxS | 0.49 | -0.08 | 0.95 | 0.137 |
|  | Low | 0.58 | 0.11 | 0.8 | 0.109 |
|  | N1 | 0.67 | 0.22 | 0.91 | 0.171 |
|  | N1EM | 0.43 | 0.08 | 0.28 | 0.222 |
|  | S | 0.48 | -0.57 | 0.47 | 0.226 |
| UA | All | 0.63 | -0.01 | 0.92 | 0.062 |
|  | K | 0.51 | 0.09 | 0.49 | 0.194 |
|  | KxS | 0.59 | -0.24 | 0.46 | 0.247 |
|  | Low | 0.55 | 0.18 | 0.66 | 0.111 |
|  | N1 | 0.53 | 0.47 | 0.2 | 0.213 |
|  | N1EM | 0.62 | 0.09 | 0.91 | 0.168 |
|  | S | 0.95 | -1.11 | -0.01 | 0.251 |
| YLD | All | 0.63 | 0 | 0.88 | 0.046 |
|  | K | 0.9 | 0.75 | -0.19 | 0.231 |
|  | KxS | 0.6 | -0.36 | -0.25 | 0.219 |
|  | Low | 0.75 | 0.32 | 0.31 | 0.1 |
|  | N1 | 0.57 | 0.11 | 0.2 | 0.236 |
|  | N1EM | 0.81 | 0.45 | 0.17 | 0.215 |
|  | S | 0.29 | -0.93 | 0.27 | 0.3 |
| ^1^mean squared error  ^2^average intercept of the regression  ^3^standard deviation of the accuracy across folds and replicates | | | | | |

| Table S3: Sparse Partial Least Squares Regression statistics from 20 replicates of 5-fold CV. | | | | | |
| --- | --- | --- | --- | --- | --- |
| **TRAIT** | **POP** | **MSE^1^** | **INTER^2^** | **SLOPE** | **SDEV^3^** |
| ANT | All | 0.24 | 0.01 | 0.99 | 0.025 |
|  | K | 0.08 | 0.82 | 0.04 | 0.209 |
|  | KxS | 0.32 | -0.1 | 0.57 | 0.193 |
|  | Low | 0.16 | 0.08 | 0.86 | 0.069 |
|  | N1 | 0.21 | -0.09 | 1.19 | 0.167 |
|  | N1EM | 0.19 | 0.11 | 0.58 | 0.202 |
|  | S | 0.41 | -1.4 | 0.19 | 0.327 |
| ASH | All | 0.66 | 0.01 | 0.96 | 0.064 |
|  | K | 0.51 | -0.18 | 0.39 | 0.266 |
|  | KxS | 0.75 | 0.29 | 0.74 | 0.206 |
|  | Low | 0.46 | -0.16 | 0.64 | 0.103 |
|  | N1 | 0.42 | -0.21 | 0.53 | 0.183 |
|  | N1EM | 0.47 | -0.24 | 0.64 | 0.219 |
|  | S | 1.28 | 0.21 | 0.67 | 0.21 |
| AX | All | 0.67 | 0 | 0.98 | 0.06 |
|  | K | 0.55 | 0.63 | 3.05 | 0.183 |
|  | KxS | 0.68 | 0.11 | 0.55 | 0.251 |
|  | Low | 0.66 | -0.01 | 1.03 | 0.115 |
|  | N1 | 0.82 | -0.47 | -0.3 | 0.193 |
|  | N1EM | 0.63 | 0.9 | 3.02 | 0.212 |
|  | S | 0.71 | 0.42 | 0.6 | 0.27 |
| ETOH | All | 0.61 | 0 | 0.96 | 0.057 |
|  | K | 0.5 | 0.11 | 0.64 | 0.183 |
|  | KxS | 0.62 | -0.13 | 0.82 | 0.185 |
|  | Low | 0.58 | 0.12 | 0.75 | 0.121 |
|  | N1 | 0.73 | 0.22 | 0.88 | 0.253 |
|  | N1EM | 0.5 | 0.02 | 0.77 | 0.17 |
|  | S | 0.72 | -0.55 | 0.45 | 0.234 |
| FAT | All | 0.55 | -0.01 | 0.97 | 0.047 |
|  | K | 0.44 | 0.12 | 0.69 | 0.18 |
|  | KxS | 0.79 | -0.71 | 0.3 | 0.178 |
|  | Low | 0.42 | 0.05 | 0.95 | 0.088 |
|  | N1 | 0.37 | -0.16 | 1.41 | 0.151 |
|  | N1EM | 0.41 | 0.06 | 0.91 | 0.177 |
|  | S | 0.74 | -0.8 | -0.03 | 0.238 |
| FEST | All | 0.31 | 0 | 1.01 | 0.029 |
|  | K | 0.22 | 0.43 | 0.42 | 0.204 |
|  | KxS | 0.47 | -0.02 | 0.98 | 0.208 |
|  | Low | 0.25 | 0.02 | 0.99 | 0.103 |
|  | N1 | 0.29 | 0.05 | 0.93 | 0.204 |
|  | N1EM | 0.23 | -0.13 | 1.15 | 0.179 |
|  | S | 0.35 | 0 | 1.03 | 0.178 |
| FRU | All | 0.66 | 0 | 0.93 | 0.056 |
|  | K | 0.77 | 0.3 | 0.15 | 0.209 |
|  | KxS | 0.5 | -0.04 | 0.99 | 0.194 |
|  | Low | 0.75 | 0.17 | 0.63 | 0.113 |
|  | N1 | 0.88 | 0.38 | 0.69 | 0.207 |
|  | N1EM | 0.63 | 0.08 | 0.27 | 0.208 |
|  | S | 0.53 | -0.58 | 0.38 | 0.251 |
| GLCS | All | 0.61 | -0.01 | 0.94 | 0.059 |
|  | K | 0.7 | 0.39 | 0.01 | 0.183 |
|  | KxS | 0.57 | -0.09 | 0.86 | 0.204 |
|  | Low | 0.62 | 0.26 | 0.43 | 0.099 |
|  | N1 | 0.65 | 0.45 | 0.42 | 0.192 |
|  | N1EM | 0.53 | 0.15 | 0.35 | 0.193 |
|  | S | 0.61 | -0.22 | 0.79 | 0.198 |
| GRN | All | 0.23 | -0.01 | 1.01 | 0.018 |
|  | K | 0.2 | 1.43 | -1.13 | 0.254 |
|  | KxS | 0.33 | -0.76 | 0.1 | 0.22 |
|  | Low | 0.19 | 0.15 | 0.86 | 0.12 |
|  | N1 | 0.23 | -0.64 | 2.27 | 0.178 |
|  | N1EM | 0.14 | 0.74 | -0.28 | 0.213 |
|  | S | 0.31 | -0.6 | 0.54 | 0.325 |
| HEX | All | 0.41 | -0.01 | 0.97 | 0.039 |
|  | K | 0.42 | 0.22 | 0.61 | 0.224 |
|  | KxS | 0.47 | -0.14 | 0.79 | 0.18 |
|  | Low | 0.35 | 0.31 | 0.45 | 0.101 |
|  | N1 | 0.33 | 0.35 | 0.24 | 0.22 |
|  | N1EM | 0.27 | 0.38 | 0.5 | 0.214 |
|  | S | 0.53 | -0.41 | 0.72 | 0.232 |
| HEXE | All | 0.59 | -0.01 | 0.96 | 0.06 |
|  | K | 0.49 | 0.15 | 0.6 | 0.222 |
|  | KxS | 0.65 | -0.22 | 0.77 | 0.228 |
|  | Low | 0.45 | 0.17 | 0.66 | 0.104 |
|  | N1 | 0.43 | 0.22 | 0.5 | 0.193 |
|  | N1EM | 0.44 | 0.24 | 0.63 | 0.179 |
|  | S | 0.97 | -0.32 | 0.7 | 0.212 |
| HEXEP | All | 0.67 | 0 | 0.94 | 0.061 |
|  | K | 0.57 | 0.14 | 0.46 | 0.175 |
|  | KxS | 0.68 | -0.08 | 0.76 | 0.191 |
|  | Low | 0.64 | 0.07 | 0.85 | 0.115 |
|  | N1 | 0.72 | 0.21 | 1.08 | 0.213 |
|  | N1EM | 0.64 | -0.09 | 0.83 | 0.178 |
|  | S | 0.78 | -0.69 | 0.31 | 0.216 |
| IVDMD | All | 0.55 | 0 | 0.96 | 0.053 |
|  | K | 0.44 | 0.27 | 0.53 | 0.172 |
|  | KxS | 0.57 | -0.3 | 0.57 | 0.203 |
|  | Low | 0.48 | 0.12 | 0.8 | 0.085 |
|  | N1 | 0.54 | 0.44 | 0.51 | 0.206 |
|  | N1EM | 0.47 | 0.02 | 0.59 | 0.2 |
|  | S | 0.75 | -0.89 | 0.19 | 0.172 |
| NSC | All | 0.65 | 0 | 0.94 | 0.061 |
|  | K | 0.76 | 0.32 | 0.35 | 0.185 |
|  | KxS | 0.69 | -0.18 | 0.72 | 0.212 |
|  | Low | 0.67 | 0.13 | 0.75 | 0.099 |
|  | N1 | 0.76 | 0.11 | 1.04 | 0.207 |
|  | N1EM | 0.47 | -0.01 | 0.53 | 0.222 |
|  | S | 0.54 | -0.21 | 0.74 | 0.191 |
| NSCE | All | 0.68 | -0.01 | 0.94 | 0.058 |
|  | K | 0.77 | 0.42 | 0.1 | 0.217 |
|  | KxS | 0.74 | -0.12 | 0.78 | 0.198 |
|  | Low | 0.68 | 0.1 | 0.76 | 0.093 |
|  | N1 | 0.8 | 0.28 | 0.78 | 0.189 |
|  | N1EM | 0.5 | -0.08 | 0.64 | 0.18 |
|  | S | 0.59 | -0.18 | 0.76 | 0.227 |
| PCA | All | 0.46 | 0 | 1.01 | 0.044 |
|  | K | 0.37 | 0.33 | 0.58 | 0.22 |
|  | KxS | 0.57 | -0.08 | 0.64 | 0.214 |
|  | Low | 0.37 | 0.02 | 0.96 | 0.099 |
|  | N1 | 0.41 | 0.26 | 0.28 | 0.199 |
|  | N1EM | 0.33 | -0.19 | 1.3 | 0.179 |
|  | S | 0.63 | -0.11 | 0.95 | 0.234 |
| PPEN | All | 0.49 | 0.01 | 0.96 | 0.047 |
|  | K | 0.51 | -0.23 | 0.74 | 0.209 |
|  | KxS | 0.43 | 0.2 | 0.66 | 0.157 |
|  | Low | 0.49 | -0.21 | 0.61 | 0.099 |
|  | N1 | 0.58 | -0.16 | 0.53 | 0.171 |
|  | N1EM | 0.38 | -0.29 | 0.5 | 0.209 |
|  | S | 0.51 | 0.68 | 0.48 | 0.249 |
| PSOL | All | 0.67 | 0 | 0.95 | 0.07 |
|  | K | 0.84 | 0.32 | 0.25 | 0.232 |
|  | KxS | 0.64 | -0.09 | 0.77 | 0.18 |
|  | Low | 0.75 | 0.1 | 0.76 | 0.099 |
|  | N1 | 0.9 | 0.2 | 0.99 | 0.183 |
|  | N1EM | 0.53 | -0.04 | 0.41 | 0.183 |
|  | S | 0.5 | -0.26 | 0.7 | 0.207 |
| SC | All | 0.56 | -0.01 | 0.98 | 0.048 |
|  | K | 0.68 | 0.34 | 0.41 | 0.206 |
|  | KxS | 0.5 | -0.08 | 0.93 | 0.149 |
|  | Low | 0.61 | 0.04 | 0.94 | 0.097 |
|  | N1 | 0.66 | 0.22 | 0.92 | 0.171 |
|  | N1EM | 0.46 | -0.07 | 0.58 | 0.214 |
|  | S | 0.45 | -0.27 | 0.71 | 0.245 |
| SUC | All | 0.55 | 0 | 0.97 | 0.05 |
|  | K | 0.69 | 0.42 | 0.16 | 0.182 |
|  | KxS | 0.5 | -0.07 | 0.91 | 0.142 |
|  | Low | 0.59 | 0.11 | 0.81 | 0.113 |
|  | N1 | 0.67 | 0.13 | 1.06 | 0.167 |
|  | N1EM | 0.42 | 0.04 | 0.37 | 0.237 |
|  | S | 0.5 | -0.74 | 0.31 | 0.222 |
| UA | All | 0.62 | 0 | 0.92 | 0.063 |
|  | K | 0.53 | 0.15 | 0.25 | 0.218 |
|  | KxS | 0.59 | -0.23 | 0.47 | 0.231 |
|  | Low | 0.55 | 0.15 | 0.71 | 0.105 |
|  | N1 | 0.5 | 0.4 | 0.32 | 0.204 |
|  | N1EM | 0.6 | 0.04 | 0.95 | 0.12 |
|  | S | 0.96 | -1.03 | 0.07 | 0.232 |
| YLD | All | 0.58 | 0 | 0.99 | 0.043 |
|  | K | 0.87 | -1.22 | 4.25 | 0.241 |
|  | KxS | 0.46 | -0.14 | 0.56 | 0.201 |
|  | Low | 0.71 | -0.12 | 1.27 | 0.11 |
|  | N1 | 0.56 | 0.79 | -1.34 | 0.204 |
|  | N1EM | 0.73 | 0.56 | -0.1 | 0.206 |
|  | S | 0.22 | -0.64 | 0.49 | 0.309 |
| ^1^mean squared error  ^2^average intercept of the regression  ^3^standard deviation of the accuracy across folds and replicates | | | | | |

| Table S4: BayesB Regression statistics from 5-fold CV. Using 5000 iterations and a 1500 iteration burn-in period (see Methods Section). | | | | | |
| --- | --- | --- | --- | --- | --- |
| **TRAIT** | **POP** | **MSE^1^** | **INTER^2^** | **SLOPE** | **SDEV^3^** |
| YLD | all | 0.6 | 0.04 | 1.03 | 0.044 |
|  | Low | 0.66 | 0.4 | 1.16 | 0.159 |
|  | KxS | 0.55 | -0.3 | -0.14 | 0.201 |
|  | N1 | 0.52 | 0.37 | 0.71 | 0.267 |
|  | K | 0.8 | 0.49 | 1.15 | 0.309 |
|  | N1EM | 0.67 | 0.41 | 1.02 | 0.156 |
|  | S | 0.22 | -1.02 | 0.18 | 0.29 |
| ANT | all | 0.24 | 0.02 | 1.06 | 0.017 |
|  | Low | 0.19 | 0.41 | 1.25 | 0.07 |
|  | KxS | 0.29 | -0.24 | 0.61 | 0.28 |
|  | N1 | 0.24 | 0.5 | 1.58 | 0.286 |
|  | K | 0.08 | 0.76 | -0.34 | 0.297 |
|  | N1EM | 0.21 | 0.3 | 0.94 | 0.112 |
|  | S | 0.34 | -1.4 | 0.52 | 0.149 |
| GRN | all | 0.22 | 0.02 | 1.01 | 0.019 |
|  | Low | 0.21 | 0.63 | 0.12 | 0.15 |
|  | KxS | 0.3 | -0.77 | -0.64 | 0.255 |
|  | N1 | 0.24 | 0.68 | -0.34 | 0.243 |
|  | K | 0.23 | 0.61 | 1.03 | 0.137 |
|  | N1EM | 0.15 | 0.58 | -0.10 | 0.296 |
|  | S | 0.22 | 0.02 | 0.30 | 0.019 |
| IVDMD | all | 0.51 | 0 | 0.94 | 0.025 |
|  | Low | 0.47 | 0.43 | 0.93 | 0.085 |
|  | KxS | 0.56 | -0.4 | 0.85 | 0.298 |
|  | N1 | 0.51 | 0.47 | 0.85 | 0.141 |
|  | K | 0.49 | 0.61 | 0.20 | 0.234 |
|  | N1EM | 0.45 | 0.31 | 0.71 | 0.12 |
|  | S | 0.65 | -1.09 | 0.03 | 0.197 |
| FAT | all | 0.55 | -0.05 | 1.11 | 0.037 |
|  | Low | 0.43 | 0.44 | 1.05 | 0.138 |
|  | KxS | 0.79 | -0.78 | 0.61 | 0.361 |
|  | N1 | 0.44 | 0.44 | 1.19 | 0.125 |
|  | K | 0.41 | 0.34 | 0.73 | 0.2 |
|  | N1EM | 0.44 | 0.46 | 1.42 | 0.107 |
|  | S | 0.82 | -0.73 | 0.23 | 0.101 |
| ASH | all | 0.69 | -0.01 | 1.17 | 0.055 |
|  | Low | 0.51 | -0.41 | 0.33 | 0.039 |
|  | KxS | 0.79 | 0.46 | 0.76 | 0.16 |
|  | N1 | 0.38 | -0.4 | 0.52 | 0.246 |
|  | K | 0.45 | -0.33 | 0.69 | 0.266 |
|  | N1EM | 0.47 | -0.45 | 1.06 | 0.141 |
|  | S | 1.02 | 0.82 | 0.06 | 0.235 |
| UA | all | 0.62 | 0.02 | 0.99 | 0.084 |
|  | Low | 0.53 | 0.36 | 1.02 | 0.08 |
|  | KxS | 0.56 | -0.39 | 0.66 | 0.248 |
|  | N1 | 0.52 | 0.32 | 1.38 | 0.231 |
|  | K | 0.56 | 0.37 | 0.52 | 0.225 |
|  | N1EM | 0.7 | 0.44 | 1.92 | 0.249 |
|  | S | 0.59 | -1.01 | 0.34 | 0.209 |
| PCA | all | 0.41 | -0.03 | 1.01 | 0.037 |
|  | Low | 0.41 | 0.43 | 0.76 | 0.146 |
|  | KxS | 0.48 | -0.24 | 0.63 | 0.258 |
|  | N1 | 0.42 | 0.46 | 0.95 | 0.34 |
|  | K | 0.32 | 0.49 | 0.51 | 0.188 |
|  | N1EM | 0.45 | 0.4 | 1.87 | 0.121 |
|  | S | 0.55 | -1.26 | 0.5 | 0.455 |
| FEST | all | 0.32 | 0.02 | 0.96 | 0.02 |
|  | Low | 0.26 | 0.52 | 1.03 | 0.081 |
|  | KxS | 0.36 | -0.42 | 0.85 | 0.064 |
|  | N1 | 0.27 | 0.5 | 1.67 | 0.147 |
|  | K | 0.18 | 0.53 | 0.81 | 0.202 |
|  | N1EM | 0.27 | 0.44 | 0.74 | 0.176 |
|  | S | 0.38 | -1.13 | 1.09 | 0.189 |
| AX | all | 0.66 | 0 | 0.85 | 0.068 |
|  | Low | 0.7 | -0.38 | 0.14 | 0.097 |
|  | KxS | 0.63 | 0.21 | 0.70 | 0.055 |
|  | N1 | 0.72 | -0.34 | 2.17 | 0.081 |
|  | K | 0.45 | -0.41 | 0.67 | 0.057 |
|  | N1EM | 0.57 | -0.28 | 0.72 | 0.126 |
|  | S | 0.71 | 1.02 | 0.12 | 0.372 |
| SUC | all | 0.52 | 0.01 | 1.06 | 0.023 |
|  | Low | 0.51 | 0.43 | 1.14 | 0.07 |
|  | KxS | 0.49 | -0.34 | 1.39 | 0.165 |
|  | N1 | 0.64 | 0.48 | 0.37 | 0.283 |
|  | K | 0.68 | 0.5 | 0.58 | 0.112 |
|  | N1EM | 0.55 | 0.31 | -0.44 | 0.281 |
|  | S | 0.4 | -0.96 | 0.33 | 0.325 |
| GLCS | all | 0.45 | 0.05 | 1.08 | 0.031 |
|  | Low | 0.53 | 0.44 | 0.71 | 0.116 |
|  | KxS | 0.55 | -0.31 | 1.65 | 0.095 |
|  | N1 | 0.7 | 0.46 | 1.41 | 0.095 |
|  | K | 0.75 | 0.41 | 1.37 | 0.148 |
|  | N1EM | 0.74 | 0.38 | 0.39 | 0.202 |
|  | S | 0.51 | -0.89 | 0.95 | 0.181 |
| FRU | all | 0.76 | -0.02 | 1.04 | 0.096 |
|  | Low | 0.85 | 0.35 | 0.66 | 0.101 |
|  | KxS | 0.65 | -0.28 | 1.96 | 0.122 |
|  | N1 | 0.94 | 0.43 | 0.19 | 0.193 |
|  | K | 0.82 | 0.44 | -0.11 | 0.235 |
|  | N1EM | 0.55 | 0.31 | 0.53 | 0.26 |
|  | S | 0.54 | -0.88 | 0.83 | 0.043 |
| SC | all | 0.56 | -0.01 | 0.97 | 0.042 |
|  | Low | 0.57 | 0.4 | 1.17 | 0.08 |
|  | KxS | 0.56 | -0.33 | 1.25 | 0.159 |
|  | N1 | 0.63 | 0.5 | 0.56 | 0.098 |
|  | K | 0.62 | 0.54 | 1.37 | 0.101 |
|  | N1EM | 0.41 | 0.23 | 0.86 | 0.271 |
|  | S | 0.43 | -1 | 0.32 | 0.197 |
| ETOH | all | 0.62 | 0 | 0.99 | 0.038 |
|  | Low | 0.66 | 0.42 | 0.73 | 0.136 |
|  | KxS | 0.66 | -0.35 | 1.51 | 0.069 |
|  | N1 | 0.7 | 0.39 | 1.69 | 0.288 |
|  | K | 0.41 | 0.41 | 1.03 | 0.286 |
|  | N1EM | 0.65 | 0.36 | 0.88 | 0.069 |
|  | S | 0.81 | -1.01 | 0.42 | 0.28 |
| HEX | all | 0.4 | -0.01 | 1.03 | 0.047 |
|  | Low | 0.29 | 0.51 | 0.95 | 0.095 |
|  | KxS | 0.47 | -0.4 | 0.46 | 0.07 |
|  | N1 | 0.41 | 0.51 | 0.86 | 0.187 |
|  | K | 0.53 | 0.5 | 1.04 | 0.129 |
|  | N1EM | 0.26 | 0.55 | 0.46 | 0.325 |
|  | S | 0.52 | -1.11 | 0.91 | 0.179 |
| PPEN | all | 0.41 | -0.03 | 0.99 | 0.046 |
|  | Low | 0.45 | -0.44 | 1.29 | 0.044 |
|  | KxS | 0.39 | 0.43 | 0.40 | 0.293 |
|  | N1 | 0.55 | -0.43 | 1.13 | 0.118 |
|  | K | 0.48 | -0.46 | 2.23 | 0.124 |
|  | N1EM | 0.35 | -0.48 | 0.75 | 0.151 |
|  | S | 0.44 | 1.09 | 0.24 | 0.318 |
| HEXE | all | 0.61 | 0.02 | 0.91 | 0.041 |
|  | Low | 0.38 | 0.41 | 1.00 | 0.168 |
|  | KxS | 0.56 | -0.38 | 1.44 | 0.176 |
|  | N1 | 0.39 | 0.47 | 0.24 | 0.107 |
|  | K | 0.42 | 0.37 | 1.12 | 0.249 |
|  | N1EM | 0.39 | 0.47 | 0.98 | 0.219 |
|  | S | 1.1 | -1.02 | 1.91 | 0.12 |
| HEXEP | all | 0.7 | 0.02 | 0.89 | 0.04 |
|  | Low | 0.79 | 0.32 | 1.02 | 0.079 |
|  | KxS | 0.81 | -0.26 | 0.64 | 0.178 |
|  | N1 | 0.89 | 0.42 | 1.41 | 0.249 |
|  | K | 0.52 | 0.38 | 0.78 | 0.163 |
|  | N1EM | 0.73 | 0.25 | 0.82 | 0.188 |
|  | S | 0.65 | -0.94 | 0.13 | 0.144 |
| NSC | all | 0.68 | 0 | 0.96 | 0.041 |
|  | Low | 0.64 | 0.38 | 1.26 | 0.084 |
|  | KxS | 0.59 | -0.27 | 1.08 | 0.155 |
|  | N1 | 0.7 | 0.45 | 0.68 | 0.14 |
|  | K | 0.58 | 0.48 | 1.50 | 0.228 |
|  | N1EM | 0.4 | 0.23 | 0.86 | 0.19 |
|  | S | 0.64 | -1.04 | 0.21 | 0.31 |
| PSOL | all | 0.73 | -0.01 | 1.00 | 0.093 |
|  | Low | 0.68 | 0.36 | 0.85 | 0.073 |
|  | KxS | 0.61 | -0.31 | 1.22 | 0.292 |
|  | N1 | 0.96 | 0.4 | 1.20 | 0.089 |
|  | K | 0.95 | 0.51 | 0.15 | 0.127 |
|  | N1EM | 0.53 | 0.26 | 0.51 | 0.182 |
|  | S | 0.79 | -0.91 | 1.20 | 0.14 |
| NSCE | all | 0.72 | 0.01 | 1.12 | 0.071 |
|  | Low | 0.73 | 0.35 | 0.76 | 0.079 |
|  | KxS | 0.69 | -0.33 | 1.35 | 0.14 |
|  | N1 | 0.77 | 0.43 | 0.58 | 0.2 |
|  | K | 0.76 | 0.46 | 0.69 | 0.191 |
|  | N1EM | 0.55 | 0.26 | 0.56 | 0.266 |
|  | S | 0.63 | -0.81 | 0.58 | 0.291 |
| ^1^mean squared error | | | | | |
| ^2^average intercept of the regression | | | | | |
| ^3^standard deviation of the accuracy across folds and replicates | | | | | |

| Table S5: Variance components for selected traits after partitioning based on dominant principal components 1-3. | | | | |
| --- | --- | --- | --- | --- |
| **Trait** | **^a^σ_g_^2^** | **σ_e_^2^** | **σ_gA_^2^** | **σ_gW_^2^** |
| ANT | 0.223 | 0.063 | 0.172 | 0.051 |
| ASH | 0.088 | 0.106 | 0.034 | 0.054 |
| AX | 0.144 | 0.061 | 0.105 | 0.039 |
| ETOH | 0.222 | 0.051 | 0.113 | 0.109 |
| FAT | 0.074 | 0.068 | 0.038 | 0.036 |
| FEST | 0.193 | 0.098 | 0.125 | 0.068 |
| FRU | 0.189 | 0.015 | 0.122 | 0.067 |
| GLCS | 0.19 | 0.084 | 0.119 | 0.071 |
| GRN | 0.225 | 0.016 | 0.205 | 0.020 |
| HEX | 0.148 | 0.06 | 0.111 | 0.037 |
| HEXE | 0.097 | 0.077 | 0.054 | 0.043 |
| HEXEP | 0.111 | 0.054 | 0.054 | 0.057 |
| IVDMD | 0.121 | 0.036 | 0.077 | 0.044 |
| NSC | 0.245 | 0.055 | 0.11 | 0.135 |
| NSCE | 0.207 | 0.06 | 0.081 | 0.126 |
| PCA | 0.151 | 0.107 | 0.088 | 0.063 |
| PPEN | 0.207 | 0.058 | 0.14 | 0.067 |
| PSOL | 0.234 | 0.045 | 0.106 | 0.128 |
| SC | 0.238 | 0.07 | 0.122 | 0.116 |
| SUC | 0.197 | 0.061 | 0.117 | 0.08 |
| UA | 0.096 | 0.042 | 0.066 | 0.03 |
| YLD | 0.077 | 0.021 | 0.072 | 0.006 |
| ^a^Genetic variance, **σ_g_^2^**; residual variance, **σ_e_^2^**; across-population genetic variance, **σ_gA_^2^**; within-population genetic variance **σ_gW_^2^**. | | | | |

| Table S6: ANOVA of factors influencing prediction accuracy. | | |
| --- | --- | --- |
| **Source of variation** | **^a^Df** | **p-values** |
| Population | 6 | <0.0001 |
| Trait | 21 | <0.0001 |
| Method | 3 | 0.01 |
| Population x Trait | 126 | <0.0001 |
| Population x method | 18 | <0.0001 |
| Trait x Method | 63 | <0.0001 |
| Residuals | 378 |  |
| ^a^Degrees of Freedom | | |
